# Working Memory, Reasoning, and Task Switching Training: Transfer Effects, Limitations, and Great Expectations?

**DOI:** 10.1371/journal.pone.0142169

**Published:** 2015-11-10

**Authors:** Pauline L. Baniqued, Courtney M. Allen, Michael B. Kranz, Kathryn Johnson, Aldis Sipolins, Charles Dickens, Nathan Ward, Alexandra Geyer, Arthur F. Kramer

**Affiliations:** 1 Beckman Institute for Advanced Science and Technology, University of Illinois at Urbana Champaign, Urbana, Illinois, United States of America; 2 Department of Psychology, University of Illinois at Urbana Champaign, Urbana, Illinois, United States of America; 3 Aptima, Inc., Woburn, Massachusetts, United States of America; University of Groningen, NETHERLANDS

## Abstract

Although some studies have shown that cognitive training can produce improvements to untrained cognitive domains (far transfer), many others fail to show these effects, especially when it comes to improving fluid intelligence. The current study was designed to overcome several limitations of previous training studies by incorporating training expectancy assessments, an active control group, and “Mind Frontiers,” a video game-based mobile program comprised of six adaptive, cognitively demanding training tasks that have been found to lead to increased scores in fluid intelligence (G*f*) tests. We hypothesize that such integrated training may lead to broad improvements in cognitive abilities by targeting aspects of working memory, executive function, reasoning, and problem solving. Ninety participants completed 20 hour-and-a-half long training sessions over four to five weeks, 45 of whom played Mind Frontiers and 45 of whom completed visual search and change detection tasks (active control). After training, the Mind Frontiers group improved in working memory n-back tests, a composite measure of perceptual speed, and a composite measure of reaction time in reasoning tests. No training-related improvements were found in reasoning accuracy or other working memory tests, nor in composite measures of episodic memory, selective attention, divided attention, and multi-tasking. Perceived self-improvement in the tested abilities did not differ between groups. A general expectancy difference in problem-solving was observed between groups, but this perceived benefit did not correlate with training-related improvement. In summary, although these findings provide modest evidence regarding the efficacy of an integrated cognitive training program, more research is needed to determine the utility of Mind Frontiers as a cognitive training tool.

## Introduction

Cognitive training is not a new concept, despite the surge in “brain training” applications that capitalize on the marketability of programs informed by “neuroplasticity” research [1]. In any activity, prolonged experience or practice leads to proficiency in that specific process, or skilled behavior. More recently, there has been increased interest in developing training programs that lead to improvement in or “transfer” to a wider array of cognitive abilities or exercises beyond the specific task trained. In the psychology literature, this line of research is coined “cognitive training” [2–4] and is often associated with the goal to enhance cognition or ameliorate the age-related decline of cognitive abilities such as working memory, reasoning, and fluid intelligence (G*f*), abilities that have been shown to be predict performance in academic and workplace settings [5–7]. Developmental researchers also employ computerized training programs in hopes of improving cognitive abilities in children [8–13], including those from disadvantaged backgrounds [14] and those with learning difficulties [15–19].

Improvements in reasoning/G*f* have been found in several studies that employ working memory training [20, 21], task switching training [22], and reasoning training [14, 23], while improvements in working memory are primarily found in training studies that use working memory training tasks ([9, 17, 20, 24–28]). Although promising, several of these experiments, which were conducted on different age groups from children to older adults, face methodological shortcomings involving small sample sizes, single tests of cognitive transfer, and the lack of a comparable active control group [29–31]. Training-related improvement from the dual n-back working memory paradigm for example, has often not been replicated in other laboratories [32–35] (but see [36, 37]). Recent meta-analyses and reviews differ in their conclusions on the benefit of working memory training and highlight the implications of the aforementioned methodological issues [38–42]. More broadly, computer-based training paradigms, from video games to laboratory-based regimens, yield improvement in the trained tasks but limited transfer to other related abilities, including those similar to the trained tasks [14, 23, 43–50]. Thus, although behavioral and neural changes can be observed from training, these changes have not been shown to consistently translate to meaningful improvements outside of the training paradigm.

Several studies employing a multiple-task training approach, often using more complex tasks or games, show promise in engendering transfer beyond the specific trained tasks [14, 51–55] (but see [46, 48, 56–57]). To maximize training benefits in the current study, we employ working memory, reasoning, and task-switching training tasks similar to those previously mentioned, which have shown promise in enhancing working memory and reasoning/G*f*, abilities that highly overlap in the psychometric literature. We integrate six of these tasks into a mobile training platform called “Mind Frontiers,” which modifies the surface features of the training tasks (i.e., their appearance) to unify them into a Wild West-themed game. All tasks were programmed to be adaptive in difficulty, and a scoring/reward system was added to the game to promote engagement for the duration of training, which consisted of 20 hour-and-a-half-long sessions, with each game played for approximately 12 minutes.

To better attribute any training-related improvements to the Mind Frontiers program, the current study employed an active control group that also involved interaction with a mobile device and multiple adaptive training games. For the active control group, we used visual and perceptual training tasks that have been shown to produce improvements in the performance of these tasks but not improvements in working memory and reasoning/G*f* tests. This included three variants of a visual search paradigm previously used as an active control task in a working memory training study [32] and three variants of a change detection task that was shown not to transfer to untrained tasks [58].

As expectancy effects are a significant issue in cognitive training studies, we used a questionnaire to assess perceived improvement and other biases that may contribute to a placebo training effect [29, 30]. We also employed multiple transfer tests to allow analysis at the construct level and better generalize findings to improvement in cognitive abilities. We used a set of established measures from the Virginia Cognitive Aging Project Battery [59], which is comprised of tasks validated to assess key cognitive abilities including reasoning/G*f*, episodic memory, and perceptual speed. In addition, we administered neurocognitive tests to ensure comprehensive assessment of the training effects, including multiple tests of working memory, selective attention, divided attention, and task switching.

It is to be noted that while improving reasoning/G*f* abilities is a main goal of the study, we hypothesize that training with Mind Frontiers may also lead to benefits in related abilities, such as attentional control and perceptual or processing speed. As these abilities are often inter-related in the literature [59–62], we hypothesize that the Mind Frontiers group will also show improvements in “lower-level abilities” of selective attention, divided attention, and perceptual speed, especially given the speeded and game-like implementation of the tasks. Furthermore, reasoning/G*f* ability has been shown to be relatively stable in young adulthood [63–65], whereas other skills that are also recruited in reasoning/G*f* games may be more malleable or sensitive to training.

## Methods

### Participants

Participants were recruited from the University of Illinois campus and Champaign-Urbana community through flyers and online postings advertising participation in a “cognitive training study.” Pre-screening for demographic information (e.g., sex, education, English language proficiency) and game experience was administered using a survey completed over email. A few general game experience questions in the survey were embedded with other activity questions that included the Godin Leisure-Time Exercise Questionnaire [66]. More detailed information about game play experience, history and habits were queried in a post-experiment survey. Upon passing pre-screening, an experimenter followed-up with a phone interview that assessed major medical conditions that may affect neurocognitive testing. Participants eligible for the study fulfilled at least the following major requirements: (1) between 18 and 30 years old, (2) 75% right-handedness according to the Edinburgh Handedness Inventory, (3) normal or corrected-to-normal vision and hearing, (4) no major medical or psychological conditions, (5) no non-removable metal in the body, and (6) played no more than five hours per week of video games in the last six months. All participants signed informed consent forms and completed experimental procedures approved by the University of Illinois Institutional Review Board. One hundred two participants were recruited. Ninety participants completed the study and received compensation of $15/hour. Twelve individuals who dropped out or were disqualified from the study received $7.50/hour. Demographics are summarized in Table 1. More information about study procedures is available in S1 File.

**Table 1:** Demographics.

	Mind Frontiers	Active Control
Did not complete study due to various reasons	4	8
Maximum analysis *n*	45	45
Male	19	20
Age	20.8 (1.9), 18–25	21.2 (2.6), 18–28
Years of education	14.8 (1.5), 12–19	14.8 (1.7), 12–20

Study non-completion: For the Mind Frontiers group, 1 dropped out during training due to scheduling issues and 3 were disqualified during pre-testing due to exclusionary criteria. For the Active Control group, 3 were disqualified during pre-testing due to MRI exclusionary criteria, and 5 were disqualified due to scheduling difficulties during pre-testing or at the beginning of training. Shown in the second row is the number of subjects who completed the study. The following rows show demographic information for this remaining sample of participants. Mean, standard deviation (in parentheses) and range are shown for age and years of education. Age and years of education did not differ between groups (*p*>.4).

### Study Design

Participants completed three cognitive testing sessions and an MRI session before and after the training intervention. The MRI data will not be presented in this paper. Assessments were completed in a fixed order. Participants were randomly assigned to the Mind Frontiers training group or the active control training group. They completed four to five training sessions per week for four to five weeks, a total of 20 sessions; each session involved completing six cognitive training tasks (games) for approximately 12 minutes each. The task order was pseudo-randomized across sessions and all subjects completed the same order during each session. Following the training period, participants completed the same four testing sessions in reverse order. More details about the training protocol can be found in S1 File.

### Training Protocol

All participants completed training on portable handheld devices. After the first, tenth and last training sessions, participants completed a training feedback questionnaire electronically.

### Mind Frontiers

**Fig 1:**
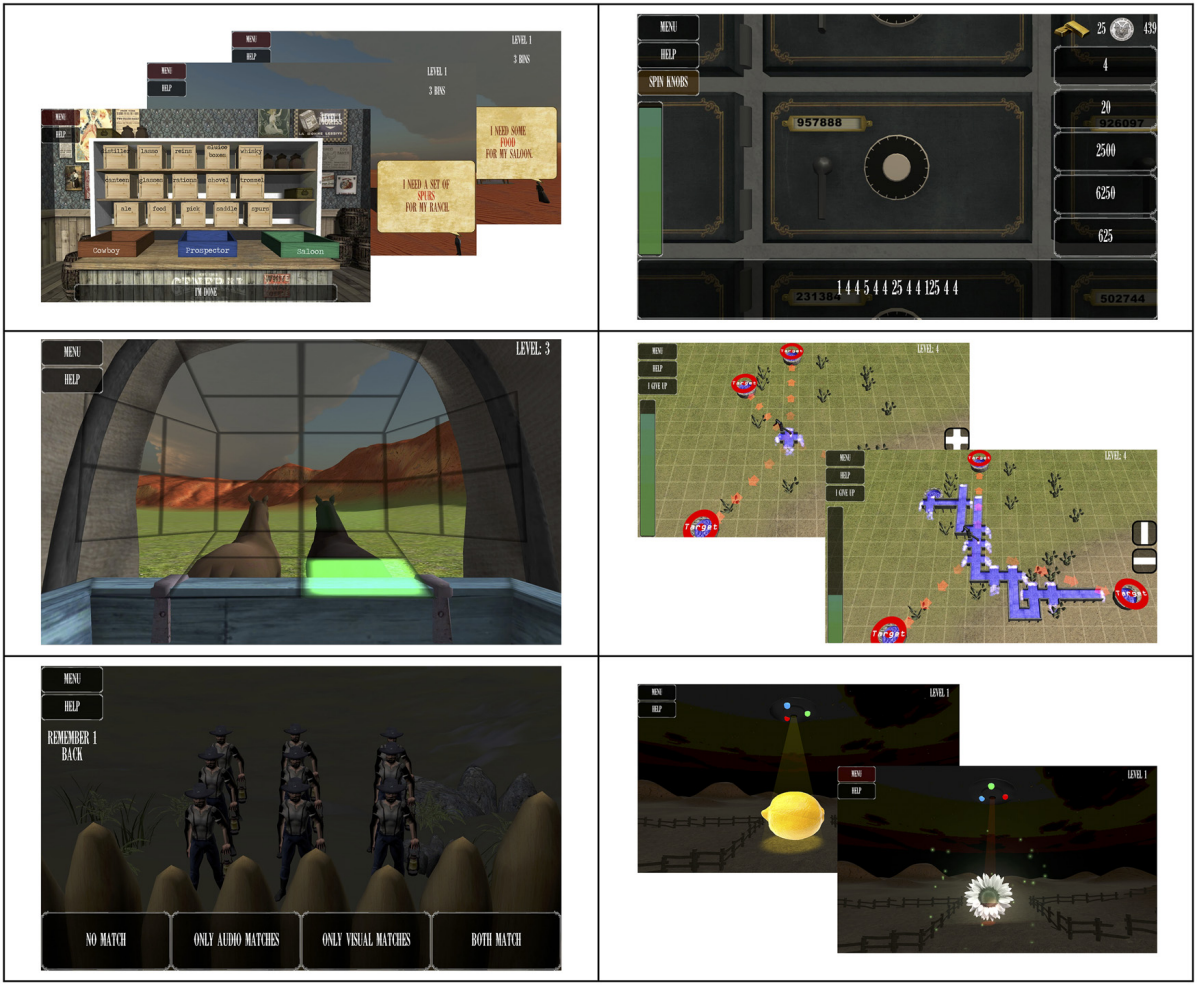
Mind Frontiers tasks. Screenshots of Mind Frontiers games: Top to bottom, left to right: Supply Run, Riding Shotgun, Sentry Duty, Safe Cracker, Irrigator, Pen ‘Em Up.

The Mind Frontiers group completed six adaptive training tasks (Table 2 and Fig 1) in each training session. All games were programmed by Aptima, Inc. using the Unity game engine and were administered using the Samsung Google Nexus 10 tablet. Table 2 provides a summary of each game and its source from previous literature. These games were selected based on their known associations (psychometric properties, training-related improvements) with the following abilities: reasoning/G*f*, working memory, visuospatial reasoning, inductive reasoning, and task switching.

**Table 2:** Training Tasks.

Game	Group	Description	Source
**Supply Run** (working memory)	Mind Frontiers	Townspeople request items that belong to a certain category. There are five objects in each category, which correspond to stereotypical occupations of the “Wild West.” Once the store is reached, the last item from each category must be selected. Difficulty level is manipulated by the number of requests and the number of categories.	Updating WM [25]
**Riding Shotgun** (working memory)	Mind Frontiers	A 20-square grid is presented. Boxes of the grid light up in a random sequence. The sequence must be entered exactly. Difficulty is manipulated by the length of the sequence.	Visuo-spatial WM [16, 20]
**Sentry Duty** (working memory)	Mind Frontiers	Sentries lift their lanterns while saying a word of the phonetic alphabet. The current word spoken and lantern lifted is compared to the word spoken/lantern lifted *n* times previously. There may be an audio match, a visual match, an audio and visual match, or no match between the current sentry and the one who spoke *n* times ago. Difficulty is manipulated by how far back the comparison is (e.g., 1-back, 2-back, 3-back).	Dual n-back [21, 32]
**Safe Cracker** (reasoning)	Mind Frontiers	Safe combinations are determined by completing the next item in a series. Series may be letter-, number-, or day/month- based, and all are governed by some pattern or rule that must be determined and applied to select the next item in the series. Difficulty is manipulated by the difficulty of the patterns and the number of problems to solve within the given time limit.	Inductive Reasoning [23]
**Irrigator** (reasoning)	Mind Frontiers	Irrigation pipelines are built from a water source to wells using individual pieces of pipe. The pipe pieces available for building are randomly determined, highlighting the importance of planning and flexibly using the resources at hand. Difficulty is manipulated by the number of wells, the presence of obstructions, and the time limit.	Visuospatial reasoning [14]
**Pen ‘Em Up** (task-switching)	Mind Frontiers	Items are presented that need to be sorted based on one of two binary criteria (for example, the item’s category or the size of the image). The pattern in which to sort the items is presented at the beginning of the level. For instance, the pattern may be to alternate sorting by category and by size. Items are sorted by swiping them either to the right or to the left. Difficulty is manipulated by increasing the complexity of the sorting pattern.	Switching without external cues [22]
**Visual Search**	Active Control	A target is presented amidst distractors. The target must be identified, and the direction of the target indicated (right/left). Difficulty is manipulated by increasing the number and heterogeneity of distractors.	[32]
**Change Detection**	Active Control	A 3- or 5- item array of stimuli is displayed. After a brief static screen (interference), the array of stimuli is presented again with one stimulus changed. The item that changed must be selected. Difficulty is manipulated by the time available to observe the initial array.	[58]

### Active Control

**Fig 2:**
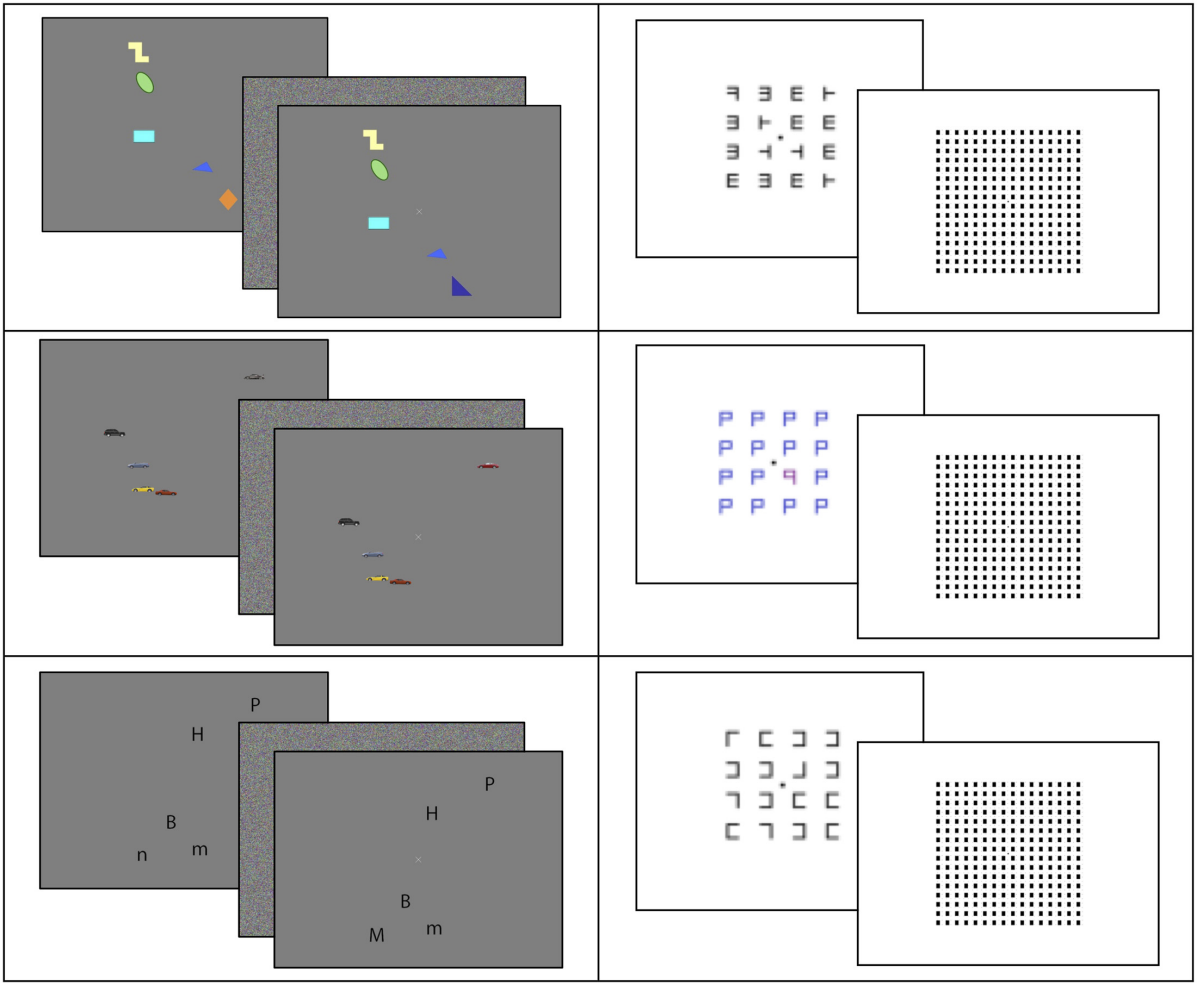
Active control tasks. Left: Screenshots of three versions of the change detection task, from top to bottom: colored shapes, cars, letters. Right: Screenshots of three versions of the visual search tasks, from top to bottom: original visual search in Redick et al. [32], colored Ps, Ls. For publication purposes, stimuli are not drawn to scale (enlarged).

The active control group also completed six adaptive training tasks in each training session (Table 2 and Fig 2). These included three variants of a visual search task and three variants of a change detection task. The visual search paradigm was derived from Redick et al. [32] and has been shown to not highly overlap (i.e., low correlations) with the working memory, reasoning, and task-switching abilities trained in Mind Frontiers [67, 68]. The change detection paradigm was obtained from Gaspar et al. [58]. Similar to the Mind Frontiers group, the active control group also completed the tasks on a portable device, the Asus Vivotab RT. The visual search tasks were programmed in E-prime 2.0 [69] and the change detection tasks were programmed in MATLAB (MathWorks™) using the Psychophysics Toolbox extensions [70, 71].

### Training Feedback Questionnaire

At the end of the first, tenth, and twentieth sessions, all participants were asked the following questions about each training game and were instructed to respond on a scale of 1–10: 1) How much did you enjoy/like each game? (1 = did not enjoy/like at all, 10 = enjoyed a lot), 2) How engaging was each game? (1 = least, 10 = greatest), 3) How demanding/effortful was each game? (1 = least, 10 = greatest), 4) How motivated were you to achieve the highest possible score on each game? (1 = least, 10 = greatest), and 5) How frustrating did you find the game? (1 = not at all frustrating, 10 = very frustrating).

### Cognitive Assessment Protocol

Before and after 20 training sessions, participants completed a battery of tests and questionnaires to assess cognitive function at pre-test and changes that may have resulted from training. The tests measured a variety of cognitive abilities, including reasoning/G*f*, episodic memory, perceptual speed, working memory, and attention (Table 3). Participants also completed questionnaires regarding sleep, personality, fitness, and media usage. Following the final testing session, participants completed a post-experiment survey that assessed their feedback on the cognitive training games, the strategies employed during training, gaming experience, and expectations. The majority of the transfer tasks have been extensively used in the cognitive psychology literature (Table 3), so only brief descriptions are provided. More details about each task can be found in S1 File.

**Table 3:** Transfer Tests.

	Category	Order	Session	Source
Shipley abstraction	Reasoning/G*f*	5	1	[72]
Matrix reasoning	Reasoning/G*f*	8	1	[73, 74]
Paper folding	Reasoning/G*f*	9	1	[75]
Spatial relations	Reasoning/G*f*	10	1	[76]
Form boards	Reasoning/G*f*	11	1	[75]
Letter sets	Reasoning/G*f*	12	1	[75]
Digit symbol substitution	Perceptual speed	1	1	[77]
Pattern comparison	Perceptual speed	2	1	[78]
Letter comparison	Perceptual speed	3	1	[78]
Logical memory	Episodic memory	4	1	[79]
Paired associates	Episodic memory	6	1	[80]
i-Position	Episodic memory	13	1	[81, 82]
Running span	Working memory	15	2	[83]
Operation span	Working memory	17	2	[84, 85]
Symmetry span	Working memory	21	2	[86]
Visual short-term memory	Working memory	24	3	[87, 88]
N-back	Working memory	19	2	[89, 90]
Dual N-back	Working memory	27	MRI	[91, 92]
Trail making	Divided attention	7	1	[93]
Attentional blink	Divided attention	14	2	[94]
Dodge	Divided attention	25	3	[95]
Flanker	Selective attention	16	2	[96, 97]
Anti-saccade	Selective attention	18	2	[96, 98, 99]
Psychomotor vigilance task	Selective attention	20	2	[96, 100]
Multi-source interference task	Selective attention	28	MRI	[101, 102]
25 boxes	Visual search	23	3	[103]
Task-switch, dual-task	Multi-tasking	22	3	[104]
Control tower	Multi-tasking	26	3	[32]

### Reasoning, perceptual speed, episodic memory

Except for i-Position, the tests below were obtained from the Virginia Cognitive Aging Project Battery [59], and two different versions were used for pre- and post-testing, with the sequence counterbalanced across subjects.

*Shipley Abstraction*: Identify missing stimuli in a progressive sequence of letters, words, or numbers. Number of correctly answered items within five minutes is the primary measure.

*Matrix Reasoning*: Select the pattern that completes a missing space on a 3 x 3 grid. Number of correctly answered items is the primary measure. Reaction time on correct trials was also analyzed.

*Paper Folding*: Identify pattern of holes that results from a punch through folded paper. Number of correctly answered items is the primary measure. Reaction time on correct trials was also analyzed.

*Spatial Relations*: Identify 3D object that would match a 2D object when folded. Number of correctly answered items is the primary measure. Reaction time on correct trials was also analyzed.

*Form Boards*: Choose shapes that will exactly fill a space. Number of correctly answered items is the primary measure.

*Letter Sets*: Determine which letter set is different from the other four. Number of correctly answered items is the primary measure. Reaction time on correct trials was also analyzed.

*Digit Symbol Substitution*: Write corresponding symbol for each digit using a coding table. The primary measure is number of correctly answered items within two minutes.

*Pattern Comparison*: Determine whether pairs of line patterns are the same or different. The primary measure is number of correctly answered items within 30 seconds, averaged across two sets of problems.

*Letter Comparison*: Determine whether pairs of letter strings are the same or different. The primary measure is number of correctly answered items within 30 seconds, averaged across two sets of problems.

*Logical Memory*: Listen to stories and recall them in detail. The primary measure is number of correctly recalled story details, summed across three story-tellings.

*Paired Associates*: Listen to word pairs and recall the second word in a pair. The primary measure is number of correctly recalled items.

*i-Position*: View an array of images on a computer screen and reproduce the positions of the images. Measures are proportion of swap errors (primary) and mean misplacement in pixels.

### Working memory

*Running Span*: Recall the last *n* items presented in a letter list that ends unpredictably. The total number of items in perfectly recalled sets is the primary measure. We also analyzed the total number of items recalled in the correct serial order, regardless of whether the set was perfectly recalled.

*Operation Span*: Remember a sequence of letters while alternately performing arithmetic problems, then recall the sequence of letters. The total number of items in perfectly recalled sets is the primary measure. We also analyzed the total number of items recalled in the correct serial order, regardless of whether the set was perfectly recalled.

*Symmetry Span*: Remember a sequence of locations of squares within a matrix while alternately judging symmetry, then recall order and locations of the sequence. The total number of items in perfectly recalled sets is the primary measure. We also analyzed the total number of items recalled in the correct serial order, regardless of whether the set was perfectly recalled.

*Visual Short-Term Memory (VSTM)*: Detect color change in an array of colored circles. Data was analyzed in terms of d-prime collapsed across set sizes (2, 4, 6, 8) and Cowan’s k averaged across set sizes [105]. Each set size measure is reported in S2 File.

*Single N-back*: Determine whether the current letter presented matches the letter presented two or three items back. The primary measure of d-prime was computed separately for the 2-back and 3-back conditions. Reaction times on correct trials were also analyzed.

*Dual N-back* (administered in the MRI): Determine whether simultaneously presented auditory and visual stimuli match stimuli presented one, two, or three items ago. The primary measure of d-prime was computed separately for the two-back and three-back conditions following procedures in [92]. Reaction times on correct trials were also analyzed.

### Divided attention, selective attention, multi-tasking

*Trail Making*: Connect numbered circles as quickly as possible by drawing a line between them in numerical order (Trails A), then connect numbered and lettered circles by drawing a line between them, alternating between numbers and letters in numerical/alphabetical order (Trails B). The difference in Trails B and Trails A completion time was the primary measure.

*Attention Blink*: Identify the white letter (target 1) in a sequence of rapidly presented black letters, and identify whether the white letter was followed by a black “X” (target 2). The attentional blink is calculated on trials where target 1 was accurately detected, as the difference in target 2 accuracy when detection is easiest (lag 8 after target 1) and when detection is most difficult (lag 2 after target 1).

*Dodge*: Avoid enemy missiles and destroy enemies by guiding the missiles into other enemies. Highest level reached within eight minutes of game play was analyzed.

*Multi-source interference task* (*MSIT*; administered in the MRI): Determine the stimulus (digits 1, 2, or 3) that is different from the other two in a three-digit number. The flanker effect is derived by taking the difference between reaction times on incongruent and congruent trials. Only correct trials were analyzed.

*Flanker*: Indicate the direction (right or left) of the middle arrow, which was either flanked by two arrows on each side (incongruent with oppositely oriented arrows, or congruent with similarly oriented arrows) or two horizontal lines on each side (neutral trials, no arrow head). The flanker effect is derived by taking the difference between reaction times on incongruent and congruent trials. Only correct trials were analyzed.

*Anti-Saccade*: Identify masked letter, cued on opposite or same side. Accuracy on a block of anti-saccade trials is used as the primary measure.

*Psychomotor Vigilance Task (PVT)*: Press key as soon as zeros begin to count up. The average of the 20% slowest RTs (bottom quintile) is used for analysis.

*25 boxes (Number Search)*: Search for stimuli in a matrix and indicate the corresponding location on blank matrix. The average score on levels with matrix rotation (levels 12–20) was analyzed.

*Control tower*: Search through arrays using different rules (primary task) while performing several distractor tasks. Performance on the primary task (average of symbol, letter and number score minus corresponding errors) was used as the main measure.

*Task-Switch*, *Dual-Task paradigm (TSDT)*: Respond to simultaneously presented auditory and visual stimuli based on cued task (auditory, visual, or both). Switch costs (reaction time difference between switch and repeat trials—for single task trials only) were analyzed separately for auditory and visual stimuli, and averaged across both.

### Self-report instruments

Participants also completed questionnaires during the third session of pre-testing. These included the Big Five Inventory [106] and Grit Scale [107] to assess personality, the Karolinska Sleep Questionnaire [108] and Pittsburgh Sleep Quality Index [109] to gauge sleep quality, the Godin Leisure-Time Exercise Questionnaire [66] to estimate physical activity, several questions on height, weight, resting heart rate and physical activity to estimate cardiorespiratory fitness [110], and a Media Multitasking Index Questionnaire [111] to assess media usage. These questionnaires were also completed post-testing, but were not used for analyses. Analyses of whether these individual differences moderate training effects will be discussed in a separate publication.

*Post-experiment questionnaire*: Participants completed an online survey that assessed gaming experience prior to and during the study, as well as their experience in the study. They provided feedback about their enjoyment, effort, and difficulty in playing the training games. They also elaborated on strategies they developed while playing the games. Participants provided feedback on game experience, design, and ease of use, and offered their perspective on improvements to their daily life resulting from their participation in the study (perceived self-improvement questions), including: overall intelligence, short-term/working memory, long-term memory, sustained attention, divided attention, visuomotor coordination, perception/visual acuity, multi-tasking, problem-solving, reasoning, spatial visualization, academic performance, emotional regulation, and work/school productivity. The fourteen dimensions queried in the perceived self-improvement questions were also posed in terms of general expectancy or perceived potential benefit. Finally, the survey assessed prior knowledge of cognitive training literature.

### Statistical Analyses

*Training tasks*: *Practice effects*: To examine improvement on the training tasks, we used a linear mixed effects model for each training task. In each of these models, the dependent variable was average level and the independent variable was session, which was coded as a linear contrast, with random effects of session and intercept for subjects. The change detection task had two conditions (set sizes three and five) which we analyzed separately.

*Training tasks*: *Composite scores*: For each training task, we computed a gain score by taking the difference between average level on the last two sessions of training and average level on the first two sessions [12, 112, 113]. To obtain a measure of overall training gain, we standardized the gain score for each relevant task and averaged the resulting values.

*Training feedback*: For each group, we averaged the training ratings across the six different tasks and analyzed each dimension using a repeated-measures ANOVA with group as between-subjects factor and training session as within-subjects factor. We report results of the multivariate tests since not all analyses met the assumption of sphericity. We do not analyze the ratings for each task, but report the means in S2 File.

*Transfer tests*: *Measures*: Primary measures for each transfer test were determined using conventional analysis procedures (S1 File). When relevant, reaction times (RTs) were also analyzed as secondary measures. In the n-back paradigm, RTs typically show a pattern that is complementary with the accuracy effects [91, 114], and each trial in both n-back training and transfer tasks required a response within a short time interval. In addition, the two reasoning games in Mind Frontiers (Irrigator, Safe Cracker) emphasized speed, such that each level needed to be completed within a limited period of time. Reasoning/G*f* tests typically have a completion time limit, but speed is usually not stressed. As strategies developed over training may be reflected in post-test performance, we also analyzed RTs for each reasoning test to determine whether training may have had a unique or differential effect on this aspect of performance.

*Transfer tests*: *Data quality and gain scores*: If participants scored more than three standard deviations from the mean of any measure (computed separately for pre- and post-test), their data was excluded from analysis of that test and its relevant composite score. This was a relatively liberal criterion applied uniformly to the measures to ensure data quality. For the letter n-back and the VSTM (only) however, this procedure identified three individuals with high d-prime values. These data points were not discarded. To reduce the influence of remaining extreme but usable values such as these, the data was then Winsorized: mean and standard deviation were recomputed for the “cleaned” dataset (separately for pre- and post-test), and any value three standard deviations away from the mean was replaced with the appropriate cut-off value (value 3 SD above the mean, or value 3 SD below the mean).

For each measure that would be analyzed at a construct level (more details in next section), we computed a standardized gain score by taking the difference between post- and pre-test scores, and dividing this by the standard deviation of the pre-test score (collapsed across groups). We also inspected gain score data quality using a more liberal criterion of four standard deviations from the mean gain score, and discarded two data points found in two subjects’ PVT gain scores (extremely negative gain scores). The task-level analysis was also not performed on the pre-subtraction measures for these excluded gain scores.

The total number of participants differed across tests due to missing or unusable data. More details regarding data quality procedures and exclusions are provided in S2 File. The raw aggregate data for each subject including outliers is provided in S3 File, together with the final data used for analyses.

*Transfer effects*: *Linear mixed model analysis*: Standardized gain scores from the transfer tests were then used for linear mixed-effects models (LME) to analyze training-related improvements at a construct level [115, 116]. A separate LME model was run for each set (i.e., construct) of gain scores, though note that not all tests were grouped into a construct. We grouped gain scores into eight constructs: working memory n-back (2-back d’, 3-back d’, Dual 2-back d’, Dual 3-back d’), working memory span (Operation Span, Running Span, Symmetry Span), reasoning/G*f* accuracy (Shipley Abstraction, Matrix Reasoning, Letter Sets, Paper Folding, Form Boards, Spatial Relations), reasoning/G*f* reaction time (Matrix, Letter Sets, Paper Folding, Spatial Relations), selective attention (Flanker, PVT, Anti-Saccade), divided attention (Dodge, Attentional Blink, Trail Making), perceptual speed (Digit-Symbol Substitution, Pattern Comparison, Letter Comparison) and episodic memory (Logical Memory, Paired Associates, i-Position). Gain scores for reaction time were multiplied by negative one, such that positive scores indicate faster performance after training. Each model consisted of a fixed effect variable of training group and crossed random effects of subject and task for the intercept [117]. These models were implemented with the “lme4” package [118]. Significance testing was performed using the standard normal distribution as well as the more conservative Kenward Rogers approximation for degrees of freedom using the “pbkr” package [119] in the R statistical program (R Core Team, 2014).

*Transfer effects*: *Composite-level analyses*: To create composite scores for use in subsequent analyses, the standardized gain scores were averaged according to the aforementioned groupings. One subject’s extremely high gain score (>4 SD) for Selective Attention was Winsorized. With these composite gain scores, we used a multivariate ANOVA to verify training group effects and their consistency with the results from the linear mixed-effects analysis. Bayes Factor was calculated using tools provided at http://pcl.missouri.edu/bf-two-sample [120].

*Transfer effects*: *Task-level analyses*: We also conducted analyses at the task level to investigate the specificity and consistency of the composite-level findings. Not all tests were integrated into a composite score or construct in the linear mixed effects analysis, and were analyzed only at the task-level. Only significant interactions at *p* < .05 are reported in the text. For brevity, we discuss significant group x time interaction results in terms of “transfer effects.” Due primarily to technical issues in the recording of responses, only 24 subjects in the Mind Frontiers group and 29 subjects in the active control group have usable dual n-back data. For each measure, we also tested whether the groups differed at baseline, and found no significant differences (S2 File).

*Perceived improvement*: Surveys with Likert-type single questions were analyzed using Mann-Whitney U tests. In S2 File, medians were used to summarize results as appropriate for ordinal data. Responses were coded as numbers prior to analysis (e.g., 1–7 for very strongly disagree to very strongly agree).

## Results

### Practice effects: Game performance across sessions

**Fig 3:**
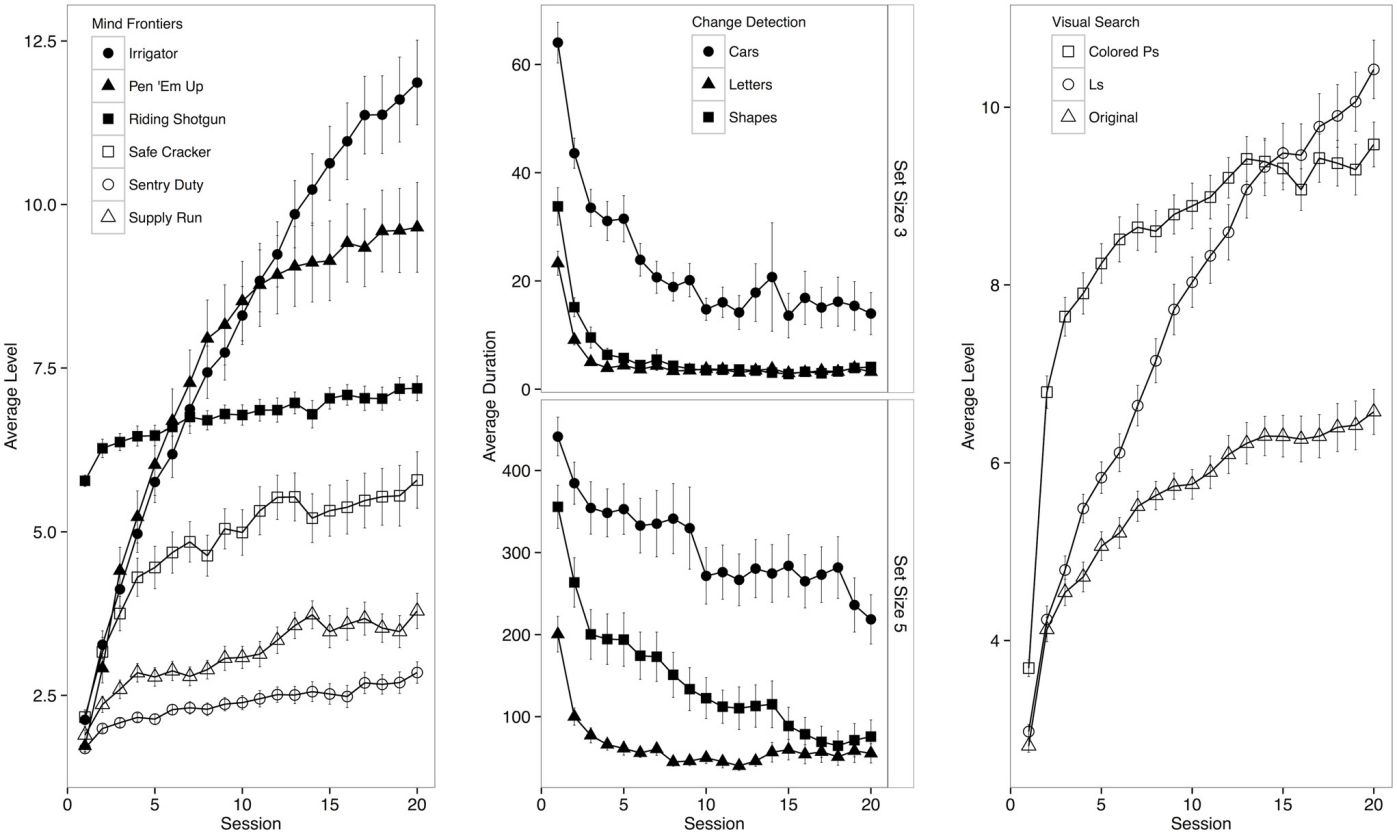
Practice effects. Panel 1: Average level across sessions for each Mind Frontiers task. Panels 2–3. Average level across sessions for each active control task. Panel 2: Change detection average maximum duration according to session and set size. Panel 3: Visual search average level according to session and task (color). Error bars are SEM.

The main effect of session was robust at *p* < .0001 for all tasks in both groups (Fig 3). The analysis of the change detection task for the set size 5 condition excluded three subjects run at the beginning of the experiment; this was due to experimenter error causing extremely high average maximum duration values in several of these subjects’ training sessions (S2 File). When these subjects were included in the set size 5 analysis, the shapes training effect was still significant (*p* = .03), the cars training effect was no longer significant (*p* = .15), and the letters training effect remained significant (*p* < .0001).

### Training Feedback

First we tested whether training feedback differed between groups after the first training session. Only motivation *F*(1,85) = 8.466, *p* = .005 and demand (*F*(1,85) = 8.858, *p* = .004 showed significant group effects, with higher overall motivation in the active control group and higher demand in the Mind Frontiers group.

We then examined whether these ratings changed over time and differed between groups. In enjoyment, there was no main effect of time, but a significant group by time interaction *F*(2,84) = 5.193, *p* = .007, *η*_*p*_^*2*^ = .110, driven by changes from the first to the tenth session. Specifically, enjoyment increased for the Mind Frontiers group and decreased for the active control group. Motivation decreased over time *F*(2,84) = 12.734, *p* < .001, *η*_*p*_^*2*^ = .233, and showed a group by time interaction *F*(2,84) = 4.580, *p* = .013, *η*_*p*_^*2*^ = .098, which was driven by greater overall motivation at session one for the active control group; there was no interaction when session one was excluded from analysis. Frustration increased mid-training, (*F*(2,84) = 6.435, *p* = .003, *η*_*p*_^*2*^ = .133) and did not differ between groups. There was no significant main effect of time and no group by time interaction in demand and engagement ratings.

Participants were not given an opportunity to rate the training tasks that they did not complete; thus the ratings provided may reflect relative differences in the six games played and not necessarily differences between training regimens. Mean ratings for each task are plotted in S2 File.

### Transfer of training: Linear mixed model analysis and composite-level analysis

As shown in Table 4, the linear mixed model analysis revealed significant transfer effects in working memory n-back and reasoning/G*f* reaction time, and a marginal effect in perceptual speed.

**Table 4:** Fixed and Random Effect Estimates for Linear Mixed Models.

	Fixed Effects						Random Effects	
Construct	Parameter	Estimate	SE	*t*	*p*.*z*	*p*.*KR*	Parameter	SD
Working memory—nback	Intercept	0.20	0.20	0.99	0.322	0.325	Task-Intercept	0.60
Working memory—nback	Group	0.72	0.20	3.66	***	***	Subject-Intercept	0.29
Working memory—nback							Residual	1.21
Working memory—span	Intercept	0.41	0.14	2.89	0.004**	0.022*	Task-Intercept	0.39
Working memory—span	Group	-0.10	0.14	-0.73	0.467	0.489	Subject-Intercept	0.18
Working memory—span							Residual	0.93
Reasoning—accuracy	Intercept	0.17	0.07	2.53	0.011*	0.016*	Task-Intercept	0.12
Reasoning—accuracy	Group	-0.03	0.10	-0.28	0.779	0.78	Subject-Intercept	0.12
Reasoning—accuracy							Residual	0.90
Reasoning—reaction time	Intercept	0.14	0.10	1.48	0.139	0.143	Task-Intercept	0.00
Reasoning—reaction time	Group	0.35	0.14	2.54	0.011*	0.013*	Subject-Intercept	0.49
Reasoning—reaction time							Residual	1.21
Selective Attention	Intercept	0.03	0.30	0.10	0.917	0.917	Task-Intercept	0.22
Selective Attention	Group	0.16	0.15	1.05	0.292	0.297	Subject-Intercept	0.03
Selective Attention							Residual	0.96
Divided Attention	Intercept	0.04	0.26	0.14	0.886	0.897	Task-Intercept	0.41
Divided Attention	Group	0.03	0.16	0.21	0.836	0.853	Subject-Intercept	0.00
Divided Attention							Residual	1.00
Perceptual Speed	Intercept	0.24	0.11	2.32	0.02	0.096	Task-Intercept	0.28
Perceptual Speed	Group	0.22	0.11	1.91	0.056	0.143	Subject-Intercept	0.41
Perceptual Speed							Residual	1.14
Episodic Memory	Intercept	0.35	0.23	1.52	0.129	0.248	Task-Intercept	0.13
Episodic Memory	Group	0.02	0.14	0.11	0.909	0.918	Subject-Intercept	0.36
Episodic Memory							Residual	1.13

*p < .05

**p < .01

***p < .001

*p*.*z* = p-value based on normal distribution, *p*.*Kr* = p-value computed with Kenward-Rogers approximation for degrees of freedom.

**Fig 4:**
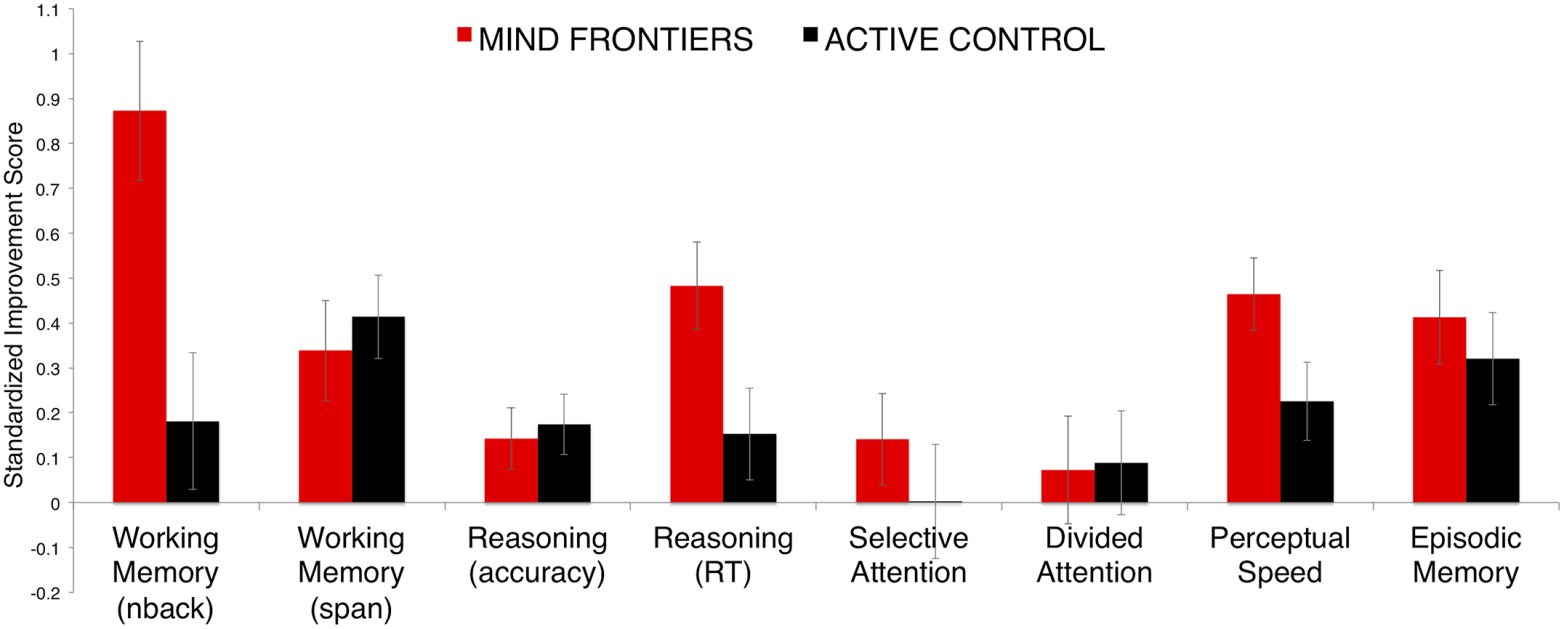
Transfer effects. Displayed are means from the MANOVA (N = 42 for Mind Frontiers, N = 45 for Active Control). Error bars are SEM.

We verified these transfer effects using the composite gain scores, which will be used in succeeding analyses. The MANOVA on the composite gain scores showed a significant training group effect *F*(8,78) = 2.633, *p* = .013, *η*_*p*_^*2*^ = .213 (Fig 4), with the pattern of results mirroring the linear mixed model analysis. The Mind Frontiers group outperformed the active control group in the working memory composite measure of n-back tests *F*(1,85) = 10.106, *p* = .002, *η*_*p*_^*2*^ = .106, which is expected given that Sentry Duty was patterned after the dual n-back. Compared to the active control group, the Mind Frontiers group also showed significantly greater gains in composite measures of reasoning/G*f* reaction time *F*(1,85) = 5.408, *p* = .022, *η*_*p*_^*2*^ = .060, and perceptual speed *F*(1,85) = 4. 007, *p* = .049, *η*_*p*_^*2*^ = .045. Due to missing data in several composite measures which overall resulted in three fewer subjects in the Mind Frontiers group, we ran separate t-tests on the composite measures and calculated Bayes Factor for each analysis. The three transfer/group effects found in the MANOVA were still significant with Bayes factor in favor of the alternative hypothesis for working memory at *t*(86) = 3.289, *p* = .001, JZS BF = 21.772, UI BF = 31.750, reasoning/G*f* RT at *t*(87) = 2.501, *p* = .014, JZS BF = 3.291, UI BF = 4.698, and perceptual speed at *t*(87) = 2.023, *p* = .046, JZS BF = 1.313, UI *BF* = 1.831. The results were not significant for all other composite measures, with JZS Bayes Factor values greater than three in favor of the null hypothesis.

In perceptual speed, the Mind Frontiers group outperformed the active control group, with the Mind Frontiers group correctly completing more items within each test’s time limit—although this effect was weaker in the linear mixed model analysis. While no group by time interaction was observed in the accuracy or total correct composite measure of reasoning/G*f*, there was a significant group x time interaction in the reaction time composite measure for reasoning/G*f*, with the Mind Frontiers group displaying faster reaction times on correct trials at post-test compared to the active controls. Working memory span, episodic memory, selective attention, and divided attention did not show training-related effects; there were no improvements or decrements that significantly differed between groups.

### Correlation between baseline performance and transfer gain

To determine whether transfer effects observed in the Mind Frontiers group vary according to baseline cognitive ability, we correlated the composite reasoning/G*f* score at baseline (pre-test) with transfer gain for the composite measures that showed a group effect. None of the correlations were significant. Since working memory ability may also predict individual differences in transfer, we correlated baseline working memory scores with transfer gain. There was no significant correlation between working memory n-back baseline score and transfer gain. Baseline working memory span score was correlated with transfer gain in perceptual speed (*r*(42) = .274, *p* = .036, one-tailed), but this is not significant after Bonferroni correction for multiple comparisons.

### Correlation between training gain and transfer gain

Next we tested whether training-related improvements related to gains observed in the transfer tests. Table 5 reports the Pearson correlation coefficients and the confidence intervals from 2000 bootstrapped samples using the adjusted bootstrap percentile (BCa) method [121]. For the Mind Frontiers training group, overall training gain was significantly related only to working memory n-back transfer gain. There was no significant relationship between training gain and the perceptual speed and reasoning RT gain scores (Table 5 and S2 File). For the active control group, no significant relationship was observed, which is not surprising given that no transfer effects were observed for this group (Table 5).

**Table 5:** Correlation between Training Gain and Transfer Gain.

	Active Control		Mind Frontiers		Supply Run	Riding Shotgun	Sentry Duty	Safe Cracker	Irrigator	Pen ‘Em Up
Transfer Gain Score	*r*	95% BCa	*r*	95% BCa	*r*	*r*	*r*	*r*	*r*	*r*
Working memory—nback	0.03	[-0.21,0.25]	0.40**	[0.10,0.61]	0.30*	0.12	0.43**	0.46**	0.21	0.2
Working memory—span	0.26	[-0.01,0.46]	0.23	[-0.13,0.49]	0.04	0.38*	0.17	0.28	0.03	0.12
Reasoning—accuracy	-0.05	[-0.36,0.21]	-0.20	[-0.44,0.04]	-0.22	-0.14	0.02	-0.28	-0.12	-0.11
Reasoning—reaction time	0.13	[-0.14,0.39]	-0.22	[-0.47,0.08]	-0.19	-0.14	-0.13	-0.13	-0.23	-0.12
Selective Attention	-0.06	[-0.33,0.24]	0.22	[0.01,0.45]	0.28	0.21	0.19	0.15	-0.05	0.18
Divided Attention	0.19	[-0.10,0.43]	-0.11	[-0.35,0.16]	-0.11	-0.02	-0.17	0.05	0	-0.2
Perceptual Speed	-0.25	[-0.50,0.06]	0.18	[-0.15,0.47]	0.21	0.1	0.18	0.1	-0.05	0.22
Episodic Memory	-0.05	[-0.29,0.19]	-0.03	[-0.29,0.26]	0.07	0	0.1	-0.19	-0.24	0.15

*p < .05

**p < .01

We also examined the relationship between transfer gain and training gain on each Mind Frontiers game, as averaging across training games may dilute task-specific effects. Consistent with the composite training gain results, working memory n-back gain was significantly related to training gains in Supply Run, Sentry Duty, and Safe Cracker. Moreover, gain in working memory span was significantly correlated with training gain in Riding Shotgun, which was based on a matrix span task. Given the number of correlations tested however, these results are not significant at *p* < .05 after Bonferroni correction for multiple comparisons.

### Correlation between training feedback and transfer gain

To determine whether subjects’ experience and involvement in the games factored into the transfer effects, we correlated the three composite scores that showed transfer effects and their ratings of the training games after the last session of training. Reported below are correlations significant at *p* < .05 and whose bootstrapped confidence intervals (2000 samples) do not include zero. First, we averaged ratings across the six Mind Frontiers games. None of the correlations were significant. Since the relationships may differ across the training games, we also conducted analyses at the task level.

The majority of the results were not robust or in the expected direction (greater gains with more positive ratings or experience), thus we refrain from interpreting them here. Reasoning RT gain was negatively correlated with demand in Sentry Duty (Kendall τ_B_ (n = 42) = -.283, *p* = .013 two-tailed, BCa 95% CI [-.484 -.069]) and with enjoyment in Pen ‘Em Up (Kendall τ_B_ (n = 42) = -.268, *p* = .016 two-tailed, BCa 95% CI [-.506 -.031]). Meanwhile, perceptual speed gain was negatively correlated with motivation in Safe Cracker (Kendall τ_B_ (n = 42) = -.251, *p* = .027 two-tailed, BCa 95% CI [-.453 -.044]). None of these correlations, however, pass a Bonferroni-corrected threshold and thus overall indicate no effect of gaming experience on transfer.

### Task-level analysis

Results for each test are summarized in Table 6 and briefly discussed below. We also tested pre-test scores and did not find significant group differences at baseline (S2 File).

**Table 6:** Transfer Results at the Task-level.

			MIND FRONTIERS		ACTIVE CONTROL	
Task	Measure	Group (2) x Session (2)	Pre	Post	Pre	Post
Dual N-Back	2-back d-prime	*F*(1,51) = 1.394, *p* = .243, ηp2 = .027	.68 (.20)	.78 (.17)	.66 (.18)	.70 (.15)
	3-back d-prime	*F*(1,51) = 8.454, *p* = .005, ηp2 = .142	.32 (.21)	.58 (.27)	.35 (.14)	.43 (.20)
	2-back RT (ms)	*F*(1,51) = 0.798, *p* = .376, ηp2 = .015	1278.11 (222.08)	1272.20 (215.49)	1408.04 (245.80)	1453.05 (212.49)
	3-back RT (ms)	*F*(1,51) = 2.267, *p* = .138, ηp2 = .043	1492.28 (333.05)	1405.65 (248.20)	1597.08 (290.28)	1629.60 (246.07)
Single N-Back	2-back d-prime	*F*(1,83) = 6.943, *p* = .010, ηp2 = .077	2.99 (.96)	3.61 (1.05)	3.14 (.92)	3.00 (1.29)
	3-back d-prime	*F*(1,83) = 4.826, *p* = .031, ηp2 = .055	1.84 (.68)	2.66 (1.21)	1.94 (.80)	2.19 (1.24)
	2-back RT (ms)	*F*(1,83) = 7.418, *p* = .008, ηp2 = .082	951.38 (155.62)	778.57 (170.28)	983.28 (134.31)	889.94 (134.43)
	3-back RT (ms)	*F*(1,83) = 15.006, *p* < .001, ηp2 = .153	1048.00 (165.84)	836.60 (199.20)	1016.64 (145.12)	953.38 (147.70)
VSTM	d-prime	*F*(1,81) = 0.556, *p* = .458, ηp2 = .007	.46 (.11)	.44 (.13)	.44 (.12)	.44 (.13)
	Cowan's k	*F*(1,81) = 0.595, *p* = .443, ηp2 = .007	1.47 (.56)	1.52 (.62)	1.28 (.56)	1.43 (.51)
Operation Span	total correct—sets	*F*(1,82) = 0.234, *p* = .630, ηp2 = .003	40.77 (19.57)	47.98 (16.42)	40.15 (15.98)	49.02 (14.48)
	total correct—items	*F*(1,82) = 0.008, *p* = .927, ηp2 < .001	55.67 (14.05)	62.72 (10.28)	56.10 (11.83)	62.93 (9.17)
Running Span	total correct—sets	*F*(1,88) = 0.839, *p* = .362, ηp2 = .009	21.80 (6.96)	22.09 (6.37)	21.16 (6.89)	22.69 (6.28)
	total correct—items	*F*(1,88) = 1.015, *p* = .316, ηp2 = .011	36.29 (9.11)	37.42 (7.38)	35.89 (8.82)	38.73 (7.79)
Symmetry Span	total correct—sets	*F*(1,82) < .001, *p* = .990, ηp2 < .001	22.51 (9.19)	27.09 (11.13)	21.85 (8.35)	26.46 (9.36)
	total correct—items	*F*(1,82) = 0.100, *p* = .753, ηp2 = .001	31.67 (6.80)	34.09 (6.85)	31.22 (6.67)	34.10 (5.88)
Matrix Reasoning	total correct items	*F*(1,85) = 5.731, *p* = .019, ηp2 = .063	10.40 (2.78)	9.81 (3.25)	9.61 (2.74)	10.50 (2.99)
	correct trial RT (ms)	*F*(1,85) = 1.553, *p* = .216, ηp2 = .018	28653.50 (4988.58)	26229.68 (6354.59)	27800.35 (7714.14)	27563.64 (7796.53)
Letter Sets	total correct items	*F*(1,84) = 0.286, *p* = .594, ηp2 = .003	12.43 (1.35)	12.67 (1.32)	12.11 (1.82)	12.11 (1.63)
	correct trial RT (ms)	*F*(1,84) = 7.691, *p* = .007, ηp2 = .084	23867.34 (6999.30)	18613.20 (5971.76)	22076.83 (6489.33)	21254.28 (7359.81)
Paper Folding	total correct items	*F*(1,87) = 0.252, *p* = .617, ηp2 = .003	8.23 (2.36)	8.77 (2.67)	8.58 (2.36)	8.91 (1.96)
	correct trial RT (ms)	*F*(1,87) = 1.275, *p* = .262, ηp2 = .014	29354.63 (11590.51)	24600.05 (10352.46)	27296.85 (11523.65)	25768.13 (12498.77)
Spatial Relations	total correct items	*F*(1,85) = 1.393, *p* = .241, ηp2 = .016	12.52 (4.35)	13.34 (4.19)	12.63 (4.10)	12.51 (4.42)
	correct trial RT (ms)	*F*(1,85) = 0.951, *p* = .332, ηp2 = .011	30174.52 (8454.12)	25880.79 (8875.31)	28534.64 (9439.90)	25889.11 (9430.65)
Form Boards	total correct items	*F*(1,85) = 1.053, *p* = .308, ηp2 = .012	10.69 (5.96)	11.57 (5.33)	9.73 (4.12)	11.51 (4.44)
Shipley Abstraction	total correct items	*F*(1,86) = 0.205, *p* = .652, ηp2 = .002	15.00 (2.77)	15.98 (2.13)	15.09 (2.71)	15.77 (1.94)
Digit Symbol Substitution	total correct items	*F*(1,85) = 2.466, *p* = .120, ηp2 = .028	94.96 (12.76)	99.71 (14.13)	90.02 (13.27)	98.30 (12.61)
Pattern Comparison	total correct items	*F*(1,86) = 3.144, *p* = .080, ηp2 = .035	19.01 (3.43)	20.90 (3.35)	18.60 (3.05)	19.25 (3.77)
Letter Comparison	total correct items	*F*(1,86) = 7.882, *p* = .006, ηp2 = .084	12.91 (2.15)	13.84 (2.17)	13.13 (2.08)	12.97 (2.04)
Logical Memory	total correct items	*F*(1,87) = 1.069, *p* = .304, ηp2 = .012	45.77 (8.01)	52.66 (8.59)	45.40 (8.72)	50.38 (9.50)
Paired Associates	total correct items	*F*(1,83) = 0.096, *p* = .758, ηp2 = .001	6.65 (3.04)	7.79 (3.31)	7.62 (3.45)	8.98 (2.54)
i-Position	swap error	*F*(1,81) = 0.189, *p* = .665, ηp2 = .002	.025 (.022)	.022 (.034)	.025 (.027)	.026 (.033)
	mean misplacement (pixels)	*F*(1,81) = 0.727, *p* = .396, ηp2 = .009	130.88 (38.99)	127.78 (48.73)	131.47 (42.20)	138.07 (53.51)
Anti-saccade	anti-saccade accuracy	*F*(1,87) = 3.951, *p* = .050, ηp2 = .043	.63 (.18)	.68 (.19)	.64 (.14)	.62 (.20)
Flanker	flanker effect (ms)	*F*(1,79) = 2.133, *p* = .148, ηp2 = .026	88.48 (38.28)	73.73 (28.24)	91.23 (31.70)	63.12 (31.30)
	incongruent RT (ms)	*F*(1,79) = 0.231, *p* = .632, ηp2 = .003	623.23 (91.95)	593.98 (77.01)	622.89 (97.47)	602.39 (91.11)
	neutral RT (ms)	*F*(1,79) = 0.573, *p* = .451, ηp2 = .007	519.28 (61.29)	508.43 (62.95)	519.40 (72.85)	518.72 (75.91)
	congruent RT (ms)	*F*(1,79) = 1.846, *p* = .178, ηp2 = .023	534.75 (72.97)	520.25 (76.32)	531.66 (86.90)	539.26 (91.81)
PVT	bottom quintile RT	*F*(1,83) = 1.802, *p* = .183, ηp2 = .021	489.61 (83.99)	504.86 (109.06)	488.39 (75.55)	534.94 (118.10)
MSIT	congruency effect (ms)	*F*(1,81) = 6.463, *p* = .013, ηp2 = .074	242.12 (63.70)	242.40 (72.62)	252.43 (76.14)	225.61 (69.80)
	congruent RT (ms)	*F*(1,81) = 2.591, *p* = .111, ηp2 = .031	738.30 (132.60)	728.82 (128.65)	739.24 (92.71)	761.35 (108.64)
	incongruent RT (ms)	*F*(1,81) = 0.054, *p* = .817, ηp2 = .001	980.71 (156.11)	971.22 (170.08)	991.67 (119.78)	986.96 (124.45)
Number Search	late level score	*F*(1,81) = 0.047, *p* = .829, ηp2 = .001	7708.95 (1174.71)	8304.36 (575.33)	7839.88 (950.34)	8477.22 (374.76)
Dodge	maximum level	*F*(1,77) = 0.814, *p* = .370, ηp2 = .010	8.53 (.91)	9.11 (.85)	8.54 (.91)	8.86 (1.14)
Attentional Blink	lag 8–2 accuracy	*F*(1,83) = 1.730, *p* = .192, ηp2 = .020	.50 (.34)	.41 (.41)	.39 (.34)	.42(.35)
	lag 2 accuracy	*F*(1,83) = 0.089, *p* = .766, ηp2 = 001	.33 (.30)	.41 (.34)	.33 (.29)	.39 (.30)
	lag 8 accuracy	*F*(1,83) = 2.704, *p* = .103, ηp2 = .032	.82 (.21)	.82 (.19)	.72 (.27)	.81 (.18)
Trail Making	trails B—A (s)	*F*(1,77) = 5.466, *p* = .022, ηp2 = .066	28.32 (13.06)	19.70 (9.38)	23.87 (13.30)	22.07 (10.75)
	trails B time (s)	*F*(1,77) = 4.939, *p* = .029, ηp2 = .060	53.12 (15.77)	40.04 (12.82)	49.78 (15.40)	42.66 (12.61)
	trails A time (s)	*F*(1,77) = 0.417, *p* = .520, ηp2 = .005	24.80 (7.28)	20.34 (5.89)	25.91 (7.73)	20.59 (4.83)
Control Tower	primary score	*F*(1,81) = 0.916, *p* = .341, ηp2 = .011	35.08 (10.79)	40.49 (11.06)	36.07 (10.51)	39.90 (9.90)
	distractor score	*F*(1,81) = 1.044, *p* = .310, ηp2 = .013	25.81 (2.04)	26.93 (1.86)	26.35 (2.20)	27.00 (1.87)
TSDT	overall switch cost (RT)	*F*(1,81) = 0.684, *p* = .410, ηp2 = .008	53.07 (75.65)	51.23 (73.33)	62.15 (81.94)	42.45 (54.68)
	visual switch cost (RT)	*F*(1,81) = 2.601, *p* = .111, ηp2 = .031	62.26 (83.07)	71.35 (90.50)	75.74 (70.86)	48.73 (57.29)
	auditory switch cost (RT)	*F*(1,81) = 0.201, *p* = .655, ηp2 = .002	46.17 (116.52)	25.21 (105.73)	41.14 (131.54)	34.59 (90.83)

### Working memory (n-back tasks)

Compared to the active control group, the Mind Frontiers group improved significantly on three out of four accuracy measures in the dual and single n-back tests. This is not surprising given that the Sentry Duty game in Mind Frontiers is based on the dual n-back task. Although the 2-back condition in the dual n-back did not reach significance, there was a trend of higher scores in the Mind Frontiers group at post-test. Reaction time improvements were also observed in the single letter n-back task.

### Working memory (span tasks and VSTM)

While there was evidence of near-transfer to the n-back tasks in the Mind Frontiers group, no transfer effects were found in other common measures of working memory such as the Operation Span, Running Span, Symmetry Span, and VSTM tasks.

### Reasoning/Fluid Intelligence

In the Matrix Reasoning task, there was a significant group by time interaction, with the active control group showing higher accuracy at post-test compared to the Mind Frontiers group. Follow-up t-tests show that this was driven by better overall post-test performance in the active control participants. Despite the differences in means at pre-test, there was no significant group effect for this baseline measure *F*(1,85) = 1.747, *p* = .190. After factoring in pre-test accuracy with post-test accuracy performance as a dependent variable, the group effect was smaller but still significant *F*(1,84) = 4.043, *p* = .048, *η*_*p*_^*2*^ = .046. There was no significant training-related effect in Matrix Reasoning reaction times, although Matrix Reasoning RT gain was negatively correlated with Matrix Reasoning accuracy gain at *r*(85) = -.269, *p* = .006, one-tailed.

Although there were no other significant group x time interactions for reasoning/G*f* total correct measures, the Mind Frontiers group at post-test had significantly faster reaction times for Letter Sets. In the other reasoning tasks, there was a trend for faster RTs in the Mind Frontiers group, but these effects were not significant at the task-level. It is important to note that RTs are not typically used as measures for reasoning/G*f* tests. We chose to analyze RTs in this study due to the speeded nature of the reasoning training tasks included in Mind Frontiers.

### Perceptual Speed

The composite gain analyses revealed a significant transfer effect for perceptual speed. Task analyses show that this was driven by a significant group x time interaction in Letter Comparison, with more correct responses answered within the time limit for the Mind Frontiers group compared to the active control group. The interactions for Pattern Comparison and Digit-Symbol Substitution were not significant, but showed the same trend for improved performance in the Mind Frontiers group.

### Episodic Memory

Logical Memory, Paired Associates and i-Position showed no significant transfer effects.

### Selective Attention

Compared to active controls, the Mind Frontiers group had marginally improved accuracy in the Anti-saccade task. No transfer effects were found in the PVT, Flanker Test, and visual/number search game (25 Boxes). Although there was a significant group x time interaction in the MSIT RT congruency effect, there were no differences in the pre-subtraction measures of incongruent and congruent RTs.

### Divided Attention

The Mind Frontiers group had significantly smaller trail-making costs at post-test, reflecting faster completion times for the alternating Trails B test. There were no transfer effects in Attentional Blink (lag 8 –lag 2 accuracy) and Dodge.

### Multi-tasking and Task Switching

No training-related effects were found in Control Tower and TSDT.

### Perceived benefit effects

Mann-Whitney U tests on the perceived self-improvement questions did not reveal significant group differences (S2 File), suggesting that the transfer effects are unlikely to be influenced by perceived improvement differences across groups. However, the same set of questions phrased in terms of general expectancy or potential benefits (not necessarily applicable to self) revealed significant group differences (S2 File) with the active control group expecting better sustained attention (*U* = 741.0, *p* = .019) and perception (*U* = 751.0, *p* = .023), and the Mind Frontiers group expecting better multi-tasking (*U* = 789.50, *p* = .045), problem-solving (*U* = 632.50, *p* = .001), and reasoning (*U* = 717.0, *p* = .012) performance. After Bonferroni correction for the fourteen multiple comparisons however, only the problem-solving effect holds. There was no significant relationship between problem-solving expectancy and transfer gain. More details about training feedback and analyses can be found in S2 File.

## Discussion

Participants who played the Mind Frontiers game showed near-transfer to the single and dual n-back tasks, which were similar in design to one of the trained working memory tasks, Sentry Duty. Training-related transfer effects were also observed for composite measures of perceptual speed and reasoning/G*f* reaction times. These speed-reaction time findings support the hypothesis that varied and integrated cognitive training in Mind Frontiers can lead to improvements in “lower-level” abilities of perceptual speed and attention—which may reflect more efficient processing of stimuli to support performance in more complex tasks. Although reasoning/G*f* improvements were not found in primary accuracy measures, improvement in reasoning/G*f* reaction times provides some promise for the plasticity of this higher-level ability. It is important to note, however, that no training-related effects were observed in five out of the eight composite measures tested here, and that differential expectancy regarding the nature of training suggests some caution in the interpretation of the results.

### Transfer effects

Baseline cognitive performance as measured by reasoning/G*f* had no effect on transfer gains in the Mind Frontiers group, which suggests that the training had a relatively uniform effect on participants. This is likely due to the adaptive and relative difficulty of the training tasks, which decreased the likelihood of performance plateaus. Other computer-based training studies have found that baseline ability measures either negatively or positively predict improvements [49, 54, 122], with results varying depending on the nature of training tasks. The null effect of baseline reasoning/G*f* in the current study may also reflect lack of power or variability, though it is also possible that the heterogeneous and adaptive games employed here decreased the likelihood of floor or ceiling effects in overall training improvement.

Similar to Irrigator, Safe Cracker, and Pen ‘Em Up in Mind Frontiers, the reasoning/G*f* transfer tests required task execution within a very limited time frame. Although the perceptual speed tasks were not reasoning in nature per se, they also involved completion of as many items as possible within a certain time limit. Did experience with these time-limited games specifically drive the transfer gains observed in the speed and RT measures? Several training studies find that only those who improve on the training tasks (“responders”) also show transfer on untrained tests (e.g., [12, 113]). To test this, we correlated overall training gain and transfer gain as measured by composite scores. Only the working memory n-back composite score was significantly and reliably related to improvement in Mind Frontiers, similar to previous findings in working memory training [34]. As expected, no significant relationship was observed for the active control group.

Apart from transfer to another working memory n-back test, Thompson and colleagues [34] did not find any transfer to untrained paradigms. In the current study, which involved a larger sample size, we found some evidence for transfer in reasoning RT and speed measures. However, these performance gains were not related to training gains and hint that the benefits observed may be due to a factor or combination of factors common to the Mind Frontiers tasks and not necessarily attributable to processes such as working memory, reasoning, or attentional control. It is plausible that rather than developing these skills per se, the overarching time-limited nature of the tasks made participants better prepared for the speed-intensive tests at post-test.

No training-related effects were observed in the working memory span tests, despite the inclusion of “Riding Shotgun,” a Mind Frontiers game that is similar to a simple working memory span task (Symmetry Span) employed in a training study that found transfer to untrained span tests [28]. These incompatible results may arise from differences in training methodology; the Mind Frontiers group spent less time overall training on the span task (20 12-minute sessions) compared to Harrison et al. [28], where only span tasks were performed for the duration of 20 45-minute sessions. In addition, transfer effects may be very specific to the type of training received. Similar to the simple span training group of [28], the Mind Frontiers group did not improve in tests of complex working memory span (Operation Span, Symmetry Span). While the previous study found improvements in Running Span for both the simple and complex working memory training groups, the training and test stimuli were the same. The absence of a Running Span effect in the current study can be attributed to the specificity of stimuli—in that the Riding Shotgun game involved spatial locations while the Running Span test involved letter stimuli. Unlike the current study, the previous experiment also incorporated performance-based bonus compensation, which may have also led to differences in motivation. Nonetheless, an examination of individual game performance and transfer gain revealed a modest yet positive relationship between working memory-span gain and training gain on Riding Shotgun. While this effect no longer holds after multiple comparison correction, it is consistent with the n-back finding of transfer gains in tests similar to the training tasks, as well as other studies that find transfer to various working memory span tests after adaptive training on verbal and visuo-spatial span tasks [17, 123].

### Expectancy and placebo effects

The responses to the perceived improvement and expectancy questions were not consistent, with no significant group differences in questions of perceived self-improvement, but slight group effects when the same questions were phrased in terms of general potential improvement. These findings, however, are not necessarily contradictory in terms of expectancy biases and may instead reveal that the participants accurately assessed the properties of their training tasks. Nonetheless, this awareness has been argued to potentially lead to sub-conscious expectations and thus placebo effects] 29, 30]. Although the transfer effects were not correlated with the improvement or expectancy ratings, we cannot conclusively rule out that the benefits observed in the Mind Frontiers group reflect placebo effects to some extent. The results obtained here also highlight the importance of wording in self-report assessments, such that subtle changes in question framing may reveal different patterns of results.

Given these findings, a more careful examination of placebo effects is warranted. One approach involves comparison to a survey-based study where participants learn about specific interventions and evaluate intervention-related outcomes [124]. Another involves having participants specifically rate perceived improvement and expectancy for specific tasks [29], rather than general abilities as implemented in the current study.

### Limitations and Future Directions

The improvement observed in reasoning/G*f* is promising, but modest; effects were found in reaction time and not accuracy, which is the more established measure for estimating reasoning/G*f* [125–127]. Future research should involve administration of more sensitive accuracy measures that may better capture any subtle changes in processing efficiency. The tests used in the current study were derived from previous studies demonstrating sensitivity to age-related differences or changes. The Matrix Reasoning test used, for example, is a modified and abbreviated computerized version of the more extensive 60-item Raven’s Advanced Progressive Matrices [126]. Although easier to administer, these abridged tests may be less suitable for detecting subtle effects or changes [128, 129], especially in relatively high-functioning young adults and in the presence of practice (test re-test) effects. Moreover, it is possible that longer and more intense training, as well as conducting a study with a larger sample size, may lead to more measurable gains in higher-level abilities of reasoning/G*f* and working memory.

Although there were no significant differences in engagement and frustration between groups, an important limitation of this study is the different training experience between training groups. Group by time interactions were found in enjoyment and motivation, with increasing ratings for the Mind Frontiers group and decreasing ratings for the active control group. While the Mind Frontiers group experienced a “gamified” experience of the tasks, the active control participants completed less visually engaging laboratory tasks without explicit progress tracking, unlike the Mind Frontiers group that received information on points accrued from gameplay and game levels attained. Unfortunately, thus far, very few training/transfer studies have collected such ratings. Therefore, it is impossible to know whether previous observations of transfer effects have been confounded by subjects’ expectancies about benefits. A follow-up study should equate the active control group on these motivational aspects of training and usability, with comparable presentation and progress tracking of the control training tasks.

As this study involves relatively high-functioning young adults, future directions include investigating whether individual differences in physical fitness and personality can moderate training and transfer benefits. Physical fitness has been shown to be highly related to executive control abilities [130, 131], while personality factors have been found to play a role in training improvement [34, 128, 132, 133]. Moreover, brain volume in specific cortical and subcortical regions have been shown to predict training and transfer benefits from videogame training [134, 135]. Analyzing structural and functional brain profiles may provide further insight into why specific interventions may be more successful for certain individuals, and help characterize the overlap between training tasks and tests that show training-related transfer.

## Supporting Information

S1 File Supplemental Methods. (DOCX)

S2 File Supplemental Analyses. (DOCX)

S3 File Dataset. (XLSX)
