# Supplementary material for: Working Memory, Reasoning, and Task Switching Training: Transfer Effects, Limitations, and Great Expectations?
Source: PLoS One. 2015 Nov 10;10(11):e0142169. doi: 10.1371/journal.pone.0142169 (PMC4640538; doi:10.1371/journal.pone.0142169)
Supplement: S1 File — (DOCX) [file pone.0142169.s001.docx]

SUPPLEMENTARY METHODS

Participants: Recruiting and Scheduling

Ads and flyers were posted stating that participants will visit the lab 28 times for $15/hr (totaling around $700) over the course of 6-7 weeks. Advertised in E-Week (U of I weekly email newsletter to staff/faculty), U of I Psychology website, U of I Virtual Job Board, and Craigslist. Paper flyers were posted around campus and local vicinity.

Prescreening Survey: Basic demographic information, contained other lifestyle questions to mask exclusionary questions and to get additional information on individual differences (exercise, time spent watching TV, reading, online, etc.)

Phone Screening and Scheduling: After passing the phone screening and hearing description of the study, participants scheduled 4 pretesting sessions. A person was scheduled if and only if we had an MRI slot that fit within the participant’s availability. After scheduling this MRI session (4th session), the first three neuropsychological assessments were scheduled. Every effort was made to schedule on separate days but due to limited amount of time from the time scheduled to an available MRI slot; this could not always be accommodated. As an alternative, we allowed two neuropsychological assessments to be scheduled on the same day and made every effort to space these two sessions far apart (although some people would not come back for two separate sessions on the same day, so we stressed at least a 15 minute break should be taken between the two sessions and encouraged more time than that if possible).

Study Design: Forms and Extra Screening

On the first session, participants read and signed 1) an overall study consent form detailing experiment procedures, and 2) a separate MRI consent form detailing MRI scanning procedures. They also completed paperwork for payment processing, handedness assessment, and MRI safety. The MRI screening form was provided by the Biomedical Imaging Center at the Beckman Institute and consists of a checklist that details conditions and implants/devices that are contraindications for MRI. Tests for color and 20/20 (or corrected 20/20) vision were administered to confirm eligibility. Weight and height were obtained, after which participants underwent mock scanning (with a mock machine and recorded scanner noises) to determine comfort level and any potential concerns regarding MRI scanning. If participants gave informed consent to participate in the study, passed the eligibility tests, and felt comfortable after the mock scan, they completed the first set of cognitive tasks (pre-testing).

Training Protocol

Mind Frontiers

- Scores were obtained from online game output to a server. These data were further processed using MATLAB to extract the level information for each game during each session.
- Computer and software specs
  - Nexus 10, touch screen handheld tablet. Mind Frontiers game designed as an Android app installed on the tablet, programmed by Aptima, Inc. using the Unity game engine
    - A unique user name and password was created for each subject. Experimenters selected an option to load an online save of the game from the previous session.
    - Occasional game glitches were reported that would either cause the game to exit to the tablet home screen, freeze the tablet screen (requiring a manual restart), or shut down the tablet. When this occurred, an experimenter was called to resolve the issue. These glitches were reported to Aptima, Inc. to be corrected.
- Instructions verbally provided are below. Additional instructions were provided on the screen.
  - SAFECRACKER
    - In this game, you will see a sequence at the bottom of the screen. Your task is to identify the next item in the sequence by choosing from the answer options on the right of the screen.
    - There will be several of these puzzles in each level. To beat each level, you must correctly solve all the puzzles before time runs out. The timer is on the bottom right of the screen.
    - If you get the answer correct, the safe will open. Tap on the money inside the safe to collect it.
  - PEN ‘EM UP
    - You will sort items according to the criteria specified at the beginning of each game. Sorting the items refers to throwing them into either the right or the left pen.
    - At the beginning of the game, you will be given criteria for image and for style. ‘Image’ refers to the categories to which items will belong, while ‘style’ refers to some other attribute of the items.
    - For example, you may be told that the items’ image will be either ‘farm animal’ or ‘flower’. The style may be ‘plain’ or ‘black and white’.
    - At the beginning of the game, you will also be given a pattern in which you should sort the items.
    - If the pattern is ‘Image x1’, you will sort all items according to image (e.g., is the item a farm animal or a flower?)
    - If the pattern is ‘Image x1, Style x1’, then you alternate your sorting criteria – you sort one item by image, the next by style, then by image, then by style, and so on until all items have been presented.
    - The patterns increase in difficulty as the level increases.
  - IRRIGATOR
    - You will build a pipe from the source to the target (or targets) by choosing from and assembling pieces of pipe that are available to you.
    - Pull the desired piece from the bottom of the screen to where you would like to place it. When you are hovering over an area where the pipe can be placed, the object outline with turn green. If you have not dragged the piece to where it can be placed, it will be red.
    - You can drag around the screen to get to the targets/wells. You can also zoom in and out.
    - A single pipe can run to multiple wells.
    - Keep an eye on the clock, on the bottom left. You must get to the target(s) before time runs out!
  - SUPPLY RUN
    - You will encounter different people who ask you for supplies. Each requested item belongs in one of the categories listed at the bottom of the screen.
    - Your task is to remember only the very last item requested in each category.
    - At the end of the level, you will enter the store. You should drag a box labeled with the last requested item in each category into that category’s bin.
    - As the number of items and the number of categories increase, you will see buttons appear in the store that will move the boxes on the shelves and/or the bins to the right or to the left. Make sure you check to the right or left in order to access all the items and fill all the bins!
  - RIDING SHOTGUN
    - Different sections of the grid will light up. Your task is to remember the sequence in which these sections light up.
    - After the sequence is complete, you must enter it into the grid.
  - SENTRY DUTY
    - You will hear the sentries say words as they lift their lights. Your job will be to remember a certain number of these word/lantern combinations, and decide if the current word spoken and/or lantern lifted are the same as a certain number back.
    - For 1 back: Every time a sentry speaks and lifts his lantern, you must respond whether or not he matched the sentry one before him. There are several possibilities:
    - There could be no match between the two sentries – neither the words they said, nor the lanterns raised were the same.
    - There could be an audio match – the sentry said the same word as the previous one, but raised a different lantern.
    - There could be a visual match – The sentry raised the same lantern as the previous one, but said different word.
    - Both audio and visual could match – The sentry said the same word and raised the same lantern as the one before him.
    - As you go on, the task will get more complicated. Instead of responding to the match between the current sentry and the one before him, you will have to respond if he matches the sentry who spoke 2 back, 3 back, 4 back, and so on.

Visual Search/Change Detection Active Control group

- - - - Scores were obtained from Visual Search by manually searching the E-Prime text output file for the highest level reached in each session. These were recorded to be manually input by experimenters as the starting level the next session. Scores were obtained from the Change Detection games using a MATLAB script that used the CSV output files from the game to calculate the average duration for encoding on trials that participants got correct. These duration values were computed separately for arrays of 3 and 5 items. Experimenters manually recorded these average durations to be used as the maximum durations in the next session. Due to experimenter, hardware, and program error, some game scores were not obtained. In these cases, scores from the closest available session were used.
- Computer and software specs
  - ASUS VivoTab Smart, 10” touch screen handheld tablet. Touch screen was used for gameplay, while a Bluetooth-connected wireless keyboard was used for game setup and surveys.
  - Visual Search games were programmed in E-Prime and played using E-Run. E-Run generated a text file output to document score information.
    - Occasional game glitches were reported, typically caused by popup windows on the tablet. When this occurred, E-Run would close and generate an error message. The experimenter would obtain the last level the participant was on when the game crashed, and begin the game from that level. At the end of the session, the experimenter would then obtain the last level from the second data file.
  - Change Detection games programmed and played using MATLAB/Psychtoolbox. The program generated CSV output files to document score information.
    - Occasional game glitches were reported with a variety of causes. When this occurred, the MATLAB presentation window would close and the cause of the error would be documented in the command window. The experimenter would address the cause of the error if possible, calculate the maximum duration values to use, and begin the task again using those values. At the end of the session, the experimenter would use the second file to obtain duration values for the next session.
- Instructions verbally provided are below. Additional instructions were provided on the screen.
  - CHANGE DETECTION
    - Your task is to detect changes in briefly presented groups of objects. Start by looking at the center of the screen. When you are ready to begin, double-tap the center of the screen. Several objects will appear on the screen for a brief period of time, then the screen will be covered by static. The pictures will then reappear, but one of them will be changed. Your job is to select the picture that changed. Double-tap the picture that you believe is different. If you’re not sure which object changed, make your best guess. The goal is to respond as quickly as possible.
    - The objects will then disappear from the screen. To start the next trial, double-tap anywhere on the center of the screen.
    - One and only one object will change in each trial. Keep in mind that you are starting each trial, so you control the pace of the experiment.
  - VISUAL SEARCH
    - Your task is to find a target amidst distractions. Once you find that target, you should indicate if it was facing to the left or to the right. You can do this by tapping the screen on either the left or right side. You can tap anywhere on the screen, but it must be on the white portions, not the black portion.

Description of Adaptive Training Procedures

Visual search

- Excerpt from Redick et al., 2013: *If subjects’ accuracy was greater than or equal to 87.5% for the block, then the level increased by 1; if accuracy was less than or equal to 75%, then the level decreased by 1. Any other accuracy led to no change in the level on the next block*.
- In the current study, the last level achieved in the previous session was used as the starting level in the next training session.

Change detection

- Excerpt from Gaspar et al., 2013: *Accuracy at each set size in each session was thresholded at 75%; that is, the presentation duration (i.e. encoding time) of the original display on each trial was shortened or lengthened adaptively, using the Quest algorithm to maintain 75% accuracy.*
- In the current study, the average duration from the previous session, calculated separately for each set size, was used as the starting maximum duration for the next training session.

Safe Cracker

- Participants had one minute to answer all questions in a level, with the number of items increasing by one at each level, starting with 3 questions at Level 1. Participants level up if they get all questions correct, and level down if they get more than two questions wrong.

Supply Run

- The number of bins or categories to be remembered increases by one with each level, with a maximum of 6 categories. The list varied in length and ended unpredictably to prevent participants from ignoring the early items presented, although on average the list length was longer at higher levels. Participants level up if they get all questions correct and level down if they get more than 25% of the categories wrong.

Riding Shotgun

- The length of the sequence to be remembered increases by one at each level. Participants level up if they answer all questions correctly, and level down if they get more than two questions wrong.

Sentry Duty

- The n in n-back increased by one per level. Participants level up if they get fewer than three answers wrong and level down if they get more than five answers wrong.

Irrigator

- At each higher level, the number of wells to be targeted increased by one, coupled with a systematic increase in the number of obstacles and in the time required to complete the level. Participants level up if they reach all the targets and level down if they miss a target. The level specified the total area covered by obstacles. See below for more information.
- Sample levels:

| Level | Targets | Obstruction Area | Time Per Target (s) | Total Time |
| --- | --- | --- | --- | --- |
| 1 | 1 | 0 | 40 | 40 |
| 5 | 5 | 10 | 30 | 150 |
| 10 | 10 | 35 | 25 | 250 |
| 15 | 15 | 70 | 19 | 285 |
| 20 | 20 | 110 | 10 | 200 |
| 25 | 20 | 135 | 10 | 200 |
| 30 | 20 | 150 | 10 | 200 |

Pen 'Em Up

- At each level, the sorting order got progressively more complicated until it was an entirely random 14-item sequence to be remembered. If participants answer fewer than two questions incorrectly, they level up. They level down if they answer more than four questions incorrectly.

Cognitive Assessment Protocol: Additional Task Information

Shipley Abstraction (paper-pencil): Participants completed one practice trial and were given 5 minutes to complete the test, which consisted of 20 items.

Matrix Reasoning (E-prime): Participants completed 2 practice trials and were given 10 minutes to complete the test, which consisted of a maximum of 18 experimental trials.

Paper Folding (E-prime): Participants were given 10 minutes to complete a maximum of 12 trials.

Spatial Relations (E-prime): Participants were given 10 minutes to complete a maximum of 20 trials.

Form Boards (E-prime): Participants were given 8 minutes to complete a maximum of 24 trials.

Letter Sets (E-prime): Participants viewed 2 practice trials and were given 10 minutes to complete a maximum of 15 trials.

Digit Symbol Substitution (paper-pencil): Participants completed 7 practice items and were given two minutes to complete a maximum of 133 items.

Pattern Comparison (paper-pencil): Participants completed 3 practice items and given 30 seconds to a complete each set of 30 items. 2 sets were administered.

Letter Comparison (paper-pencil): Participants completed 3 practice items and given 30 seconds to a complete each set of 21 items. 2 sets were administered.

Logical Memory (verbal/paper-pencil recording by experimenter): Participants listened to two story-tellings and after each storytelling were asked to recall as many details as they can. There are a total of 25 story units for each storytelling, with a point given for each correctly recalled detail. They later completed a delayed recall of the first story, without hearing a story-telling first.

Paired Associates (verbal/paper-pencil recording by experimenter): Participants are read 2 lists, with each list consisting of 6 word pairs.

i-Position (Presentation): This task was slightly modified from Monti et al. (2014). Participants were given the following instructions: “We will now do a short memory task on the computer. In this task you try to remember the location of five objects on the screen (line drawings). We will only be using the mouse for this experiment. When you first see the objects, you will click on each one and count aloud as you click “1, 2, 3” and so on. The objects will then disappear for a brief period of time, and will reappear aligned near the top of the screen. Using the mouse, click on an object and drag it to where you believe it was on the screen. Do this until you’ve placed the objects as close to their original location as you can, then press the spacebar when you are finished. Don’t worry if this isn’t entirely clear right now, we will do a short practice so you get the hang of it. Do you have any questions?” The five objects were line drawings. The delay between study and test was 4 seconds while the inter-trial interval was 2 seconds. Participants completed 3 practice trials and 15 experimental trials.

Running Span (E-prime): 500-ms per item version from the Attention & Working Memory Lab at Georgia Tech (Broadway & Engle, 2010).

Operation Span (E-prime): Version from the Attention & Working Memory Lab at Georgia Tech (Unsworth, Heitz, Schrock & Engle, 2005). We excluded data from a participant if they made more than 14 errors.

Symmetry Span (E-prime): Version from the Attention & Working Memory Lab at Georgia Tech (Unsworth, Heitz, Schrock & Engle, 2005; Redick et al., 2012). We excluded data from a participant if they made more than 10 errors.

Visual Short Term Memory Task (Matlab/Psychtoolbox): Participants completed 300 color change detection trials. We used a version detailed in Keshvari, Van den Berg & Ma (2013): http://www.ploscompbiol.org/article/info%3Adoi%2F10.1371%2Fjournal.pcbi.1002927.

Single n-back (E-prime): Task was adapted from Kane et al. (2007). Letters were presented one at a time, and participants indicated whether the current letter is the same or different as the letter presented 2 or 3 items back. They completed guided examples of a 2-back sequence, one 2-back practice block followed by a block of 2-back experimental trials, and guided examples of a 3-back sequence, and a 3-back practice block followed by a block of 3-back experimental trials. This was followed by alternating 2-back and 3-back blocks (4 more of each). The first guided example introduced the task and presented the letters in real time, without requiring a response from the participant. The next guided example walked participants through another sequence of letters, this time with responses required on each trial. In the practice blocks, feedback was provided for each trial. The practice trials consisted of 13 letters with 11 required responses for the 2-back practice, and 10 required responses for the 3-back practice. No feedback was provided for the 5 experimental blocks. 100 stimuli drawn from a set of 10 letters (mhbjkcfdgl) were presented in the 5 experimental blocks: 20 letters in each block, requiring 18 responses in each 2-back block and 17 responses in each 3-back block. Each letter was presented for 2000ms followed by a 1000 ITI blank screen. Participants were instructed to respond quickly and accurately. Stimuli were in white, bold, Courier New 44pt font presented centrally against a black background.

Dual n-back (E-prime): Task parameters follow the version used in the fMRI study of Jaeggi and colleagues (2007). Participants completed 13 blocks, starting and ending with a zero-back block, and alternating between an n-back block and a zero-back block in between. The zero-back blocks consisted of 10 trials of simultaneously presented auditory and visual stimuli. The 6 n-back blocks were chosen at random from a set of two 1-back, two 2-back and two 3-back blocks, with each n-back block consisting of 20 trials (20 simultaneously presented auditory and visual stimuli). Instructions were provided at the beginning of each block, to indicate the n-back condition and remind participants of the response mapping. Before performing the task in the MRI environment as described above, participants practiced the task outside of the scanner, completing 10 trials of each of n-back condition. In computing d-prime accuracy, target trials were defined as trials in which a response was required (match) to both auditory and visual stimuli, or to a single auditory or visual stimuli, and hits were defined as trials in which the participant responded to both types of stimuli if both were matches, or responded to an auditory or visual stimulus if the pertinent stimulus was a match. False alarms were defined as trials in which the subject emitted a response when no stimulus was a match. RTs were filtered to 200-3000 ms.

Attentional Blink (E-prime): Participants completed a block of 20 trials where they were asked to identify the white letter (B, G, or S), followed by a block of 20 trials where they were asked to determine whether the black X was present. This was followed by two dual-task blocks of 48 trials each, where they were asked to report both the identity of the white letter, and whether or not the black X was present in the letter stream.

Dodge (Flash Player): Participants practiced on level 1 (un-timed). After level 1 completion, they started on level 2 and played for 8 minutes. They were given the following instructions: “In this task you will use the arrow keys to respond. Enemies fire missiles, which will follow your ship. Avoid getting hit by enemies’ missiles and guide them into other enemies to destroy them. You will advance to the next level once you’ve destroyed all enemies. Do you have any questions?” Participants used a local, modified version that allowed for logging of game events, scores and keystrokes.

Multi-source Interference Task (E-prime): Task parameters follow Fitzgerald et al. (2010). Participants completed four runs of the MSIT (out of a set of five possible runs), with 24 congruent, 24 incongruent and twelve fixation trials in each run. Before performing the task in the MRI environment as described above, participants practiced the task outside of the scanner that involved completing five congruent, five incongruent, and three fixation trials. For the MSIT, only subjects with overall accuracy of above 70% on both incongruent and congruent conditions were included in the analysis. Only MSIT trials with reaction times between 150 and 2000 ms were analyzed.

Flanker (E-prime): Task parameters follow Unsworth and Spillers (2010). Participants completed a practice block of five trials, followed by an experimental block of 150 trials, evenly split among congruent, incongruent, and neutral trials (horizontal lines only, no arrow head), with equal numbers of left and right pointing middle arrows. All stimuli appeared above central fixation.

Anti-Saccade (E-prime): Task parameters follow Unsworth and Spillers (2010) and Kane et al. (2001). In this study, participants completed a practice block of ten trials to practice stimuli-response mapping: B-index finger, P-middle finger, R-ring finger. Participants then complete ten pro-saccade practice trials, followed by ten anti-saccade practice trials, and finally a block of 40 anti-saccade trials. Auditory feedback was provided after each practice trial, but not for the 40 experimental trials.

Psychomotor Vigilance Task (E-prime): Task parameters follow Unsworth and Spillers (2010). Participants completed eight practice trials, followed by ten minutes of experimental trials.

25 Boxes (Flash Player): Participants completed two games, each with 20 levels. They were given the following instructions: “In this task, please read the instructions on the screen, ignore the bit about the high score and let me know when you are done reading.” AFTER READING: “The targets will be specified on the upper left of the window. You can move around the grid using the arrow keys.” Participants used a local, modified version that used arrow keys to navigate and make responses. This version logged game events, scores and keystrokes.

Control Tower (Windows executable file): Two versions (A, B) from Redick et al. (2013) were used.

Task-Switch, Dual-Task Paradigm (E-prime): This task is adapted from Costa, Medeiros-Ward, Halper, Helm, and Maloney (2012): http://jov.arvojournals.org/article.aspx?articleid=2141679 and Ward et al. (In Preparation; more information available upon request). Briefly, participants receive a cue instructing them whether to perform respond to an auditory stimulus, a visual stimulus, or to both; a visual target (circle or triangle) and auditory target (“P” or “Q” sound) was presented after each cue. Participants completed a practice block of 48 trials, and five blocks of 49 trials each. Only single task trials (not dual task trials), correct trials with RTs between 200 and 2000 ms, and correct trials not preceded by errors were analyzed in the current study.
