# Supplementary material for: Working Memory, Reasoning, and Task Switching Training: Transfer Effects, Limitations, and Great Expectations?
Source: PLoS One. 2015 Nov 10;10(11):e0142169. doi: 10.1371/journal.pone.0142169 (PMC4640538; doi:10.1371/journal.pone.0142169)
Supplement: S2 File — (DOCX) [file pone.0142169.s002.docx]

SUPPLEMENTARY ANALYSES

Change detection training effects

This graph includes all subjects, including those whose data was excluded in Figure 3 due to experimenter error.

Training feedback

The colored lines show the average of each game’s ratings at the beginning, middle and end of the training, for the Mind Frontiers group (left) and the active control group (right). The average of all six training games is shown with a circle marker.

Training gain versus transfer gain

Scatterplots showing standardized training gain versus transfer gain. Shown are the 95% confidence regions for each group.

Baseline tests and reliability estimates

For each measure reported in Table 6, we tested whether the training groups differed in performance at baseline. Below are the results from one-way ANOVA tests using only subjects who completed the study. No significant differences were found. As an estimate of test reliability, we also report Pearson’s correlations between pre and post-test measures and the p-value for a one-tailed test.

| Table 6 Extended \| Baseline Tests and Reliability Estimates | | | |
| --- | --- | --- | --- |
| Task | Measure | Group (2) Effect | Test-Retest Correlation |
| Dual N-Back | 2-back d-prime | *F*(1,51)=0.147, *p*=.703 | *r*=.430, *p*=.001 |
|  | 3-back d-prime | *F*(1,51)=0.316, *p*=.577 | *r*=.444, *p*<.001 |
|  | 2-back hit RT (ms) | *F*(1,51)=4.001, *p*=.051 | *r*=.620, *p*<.001 |
|  | 3-back hit RT (ms) | *F*(1,51)=1.498, *p*=.227 | *r*=.509, *p*<.001 |
| Single N-Back | 2-back d-prime | *F*(1,83)=0.475, *p*=.492 | *r*=.225, *p*=.019 |
|  | 3-back d-prime | *F*(1,83)=0.321, *p*=.572 | *r*=.365, *p*<.001 |
|  | 2-back correct RT (ms) | *F*(1,83)=1.025, *p*=.314 | *r*=.593, *p*<.001 |
|  | 3-back correct RT (ms) | *F*(1,83)=0.862, *p*=.356 | *r*=.379, *p*<.001 |
| Visual Short-Term Memory (VSTM) | d-prime | *F*(1,81)=.480, *p*=.490 | *r*=.428, *p*<.001 |
|  | Cowan's k | *F*(1,81)=2.290, *p*=.134 | *r*=.472, *p*<.001 |
| Operation Span | total correct - sets | *F*(1,82)=.025, *p*=.874 | *r*=.560, *p*<.001 |
|  | total correct - items | *F*(1,82)=.022, *p*=.882 | *r*=.574, *p*<.001 |
| Running Span | total correct - sets | *F*(1,88)=.195, *p*=.660 | *r*=.526, *p*<.001 |
|  | total correct - items | *F*(1,88)=.045, *p*=.833 | *r*=.533, *p*<.001 |
| Symmetry Span | total correct - sets | *F*(1,82)=.118, *p*=.733 | *r*=.393, *p*<.001 |
|  | total correct - items | *F*(1,82)=.096, *p*=.758 | *r*=.487, *p*<.001 |
| Matrix Reasoning | total correct items | *F*(1,85)=1.747, *p*=.190 | *r*=.508, *p*<.001 |
|  | correct trial RT (ms) | *F*(1,85)=0.373, *p*=.543 | *r*=.274, *p*=.005 |
| Letter Sets | total correct items | *F*(1,84)=.826, *p*=.366 | *r*=.128, *p*<.121 |
|  | correct trial RT (ms) | *F*(1,84)=1.515, *p*=.222 | *r*=.357, *p*<.001 |
| Paper Folding | total correct items | *F*(1,87)=.491, *p*=.486 | *r*=.641, *p*<.001 |
|  | correct trial RT (ms) | *F*(1,87)=.705, *p*=.403 | *r*=.310, *p*=.002 |
| Spatial Relations | total correct items | *F*(1,85)=.013, *p*=.908 | *r*=.621, *p*<.001 |
|  | correct trial RT (ms) | *F*(1,85)=.729, *p*=.396 | *r*=.619, *p*<.001 |
| Form Boards | total correct items | *F*(1,85)=.768, *p*=.383 | *r*=.665, *p*<.001 |
| Shipley Abstract | total correct items | *F*(1,86)=.024, *p*=.877 | *r*=.205, *p*=.028 |
| Digit Symbol Substitution | total correct items | *F*(1,85)=3.124, *p*=.081 | *r*=.682, *p*<.001 |
| Pattern Comparison | total correct items | *F*(1,86)=.349, *p*=.556 | *r*=.541, *p*<.001 |
| Letter Comparison | total correct items | *F*(1,86)=.229, *p*=.633 | *r*=.603, *p*<.001 |
| Logical Memory | total correct items | *F*(1,87)=.044, *p*=.834 | *r*=.502, *p*<.001 |
| Paired Associates | total correct items | *F*(1,83)=1.887, *p*=.173 | *r*=.472, *p*<.001 |
| i-Position | swap error | *F*(1,81)=.002, *p*=.966 | *r*=.306, *p*=.002 |
|  | mean misplacement (pixels) | *F*(1,81)=.004, *p*=.948 | *r*=.377, *p*<.001 |
| Anti-saccade | anti-saccade accuracy | *F*(1,87)=.122, *p*=.728 | *r*=.590, *p*<.001 |
| Flanker | flanker effect (ms) | *F*(1,79)=.125, *p*=.725 | *r*=.194, *p*=.042 |
|  | incongruent RT (ms) | *F*(1,79)<.001, *p*=.987 | *r*=.588, *p*<.001 |
|  | neutral RT (ms) | *F*(1,79)<.001, *p*=.994 | *r*=.610, *p*<.001 |
|  | congruent RT (ms) | *F*(1,79)=.030, *p*=.863 | *r*=.600, *p*<.001 |
| Psychomotor Vigilance Task | bottom quintile RT | *F*(1,83)=.005, *p*=.944 | *r*=.422, *p*<.001 |
| Multi-Source Interference Task | congruency effect (ms) | *F*(1,81)=0.441, *p*=.509 | *r*=.750, *p*<.001 |
|  | congruent RT (ms) | *F*(1,81)=0.001, *p*=.970 | *r*=.698, *p*<.001 |
|  | incongruent RT (ms) | *F*(1,81)=0.130, *p*=.719 | *r*=.788, *p*<.001 |
| Number Search | late level score | *F*(1,81)=.314, *p*=.577 | *r*=.572, *p*<.001 |
| Dodge | maximum level | *F*(1,77)=.001, *p*=.972 | *r*=.153, *p*=.089 |
| Attentional Blink | lag 8 - 2 accuracy | *F*(1,83)=1.902, *p*=.172 | *r*=.429, *p*<.001 |
|  | lag 2 accuracy | *F*(1,83)=.001, *p*=.979 | *r*=.509, *p*<.001 |
|  | lag 8 accuracy | *F*(1,83)=3.611, *p*=.061 | *r*=.333, *p*<.001 |
| Trail Making | trails B - A (s) | *F*(1,77)=2.254, *p*=.137 | *r*=.375, *p*<.001 |
|  | trails B time (s) | *F*(1,77)=.908, *p*=.344 | *r*=.644, *p*<.001 |
|  | trails A time (s) | *F*(1,77)=.431, *p*=.514 | *r*=.620, *p*<.001 |
| Control Tower | primary score | *F*(1,81)=.179, *p*=.674 | *r*=.746 *p*<.001 |
|  | distractor score | *F*(1,81)=1.326, *p*=.253 | *r*=.460, *p*<.001 |
| Task-Switch Dual-Task | overall switch cost (RT) | *F*(1,81)=0.275, *p*=.601, | *r*=.072, *p*=.259 |
|  | visual switch cost (RT) | *F*(1,81)=0.628, *p*=.430 | *r*=.107, *p*=.169 |
|  | auditory switch cost (RT) | *F*(1,81)=0.034, *p*=.854 | *r*=.150, *p*=.089 |

Visual short-term memory transfer effects according to set size

Below are the VSTM transfer effects broken down by set size. There were no transfer effects (as measured below, and by a three-way interaction of set size, group and time), and no group effects at baseline.

| Table 6 Extended \| Transfer results for VSTM | | |  | | | |  | |  |
| --- | --- | --- | --- | --- | --- | --- | --- | --- | --- |
|  |  |  | | MIND FRONTIERS | | ACTIVE CONTROL | | | |
|  | Measure | Group (2) x Session (2) | | Pre | Post | Pre | | Post | |
| VSTM | set size 2 d’ | *F*(1,81)=1.714, *p*=.194, ηp2=.021 | | 2.01 (.61) | 1.81 (.64) | 1.86 (.65) | | 1.88 (.59) | |
|  | set size 4 d’ | *F*(1,81)=0.849, *p*=.360, ηp2=.010 | | 1.27 (.65) | 1.22 (.49) | 1.07 (.44) | | 1.14 (.52) | |
|  | set size 6 d’ | *F*(1,81)=0.028, *p*=.867, ηp2<.001 | | .70 (.40) | .76 (.50) | .64 (.40) | | .72 (.43) | |
|  | set size 8 d’ | *F*(1,81)=0.252, *p*=.617, ηp2=.003 | | .50 (.40) | .55 (.41) | .42 (.46) | | .53 (.34) | |
|  | set size 2 k | *F*(1,81)=1.423, *p*=.236, ηp2=.017 | | 1.28 (.28) | 1.19 (.31) | 1.19 (.34) | | 1.20 (.27) | |
|  | set size 4 k | *F*(1,81)=0.100, *p*=.752, ηp2=.001 | | 1.70 (.77) | 1.71 (.66) | 1.49 (.55) | | 1.54 (.63) | |
|  | set size 6 k | *F*(1,81)=0.059, *p*=.809, ηp2=.001 | | 1.48 (.80) | 1.61 (.98) | 1.31 (.77) | | 1.50 (.87) | |
|  | set size 8 k | *F*(1,81)=0.414, *p*=.522, ηp2=.005 | | 1.42 (1.09) | 1.59 (1.14) | 1.15 (1.21) | | 1.50 (0.93) | |

Perceived improvement results

Median responses for perceived improvement questions: “How much do you think participation in the study has changed your abilities?”

**

Expectancy or potential benefit results

Median responses for expectancy or potential benefit questions: “How much do you think the video games you played has the potential to improve the following abilities?”

Lost and excluded training data

- 1000: Data is missing for Supply Run session 17.
- 1002: Data is missing for Pen ‘Em Up session 12, and Sentry Duty & Safe Cracker session 15.
- 1004: Data is missing for Pen ‘Em Up & Supply Run session 17, and Safe Cracker session 20.
- 1009: Data is missing for Supply Run session 13.
- 1010: Data is missing for Supply Run session 5.
- 1013: Data is missing for Irrigator session 3.
- 1015: Data is missing for Safe Cracker session 8.
- 1036: Data is missing for Irrigator session 15.
- 1046: Data is missing for Supply Run session 7.
- 1048: Data is missing for Irrigator session 19.
- 1026: Data is missing for 2 sessions.
- 1030: Data is missing for 1 session.
- 1102: Data is missing for visual search (Ls version) session 7.
- 1103: Data is missing for visual search (Ls version) session 16.
- 1127: Data is missing for visual search (original version) session 8.
- 1133: Data is missing for visual search (Ls version) session 7.
- 1135: Data is missing for visual search (original version) session 5.
- 1150: Data is missing for visual search (all three versions) session 15.
- 1152: Data is missing for visual search (Ls version) session 1.
- 1152: Data is missing for visual search (Ls version) session 5.
- 1130: Data is missing for change detection setsize 3 (shape) session 19.
- 1131: Data is missing for change detection setsize 3 (letters) session 9.
- 1133: Data is missing for change detection setsize 3 (letters) session 9.
- 1134: Data is missing for change detection setsize 3 (letters) session 11.
- 1147: Data is missing for change detection setsize 3 (letters) session 14.
- 1151: Data is missing for change detection setsize 3 (shapes) session 2.
- 1102: Data is missing for change detection setsize 3 and 5 (cars) session 7.
- 1109: Data is missing for change detection setsize 3 and 5 (cars) session 8.
- 1109: Data is missing for change detection setsize 3 and 5 (letters) session 10.
- 1114: Data is missing for change detection setsize 3 and 5 (shapes) session 4.
- 1132: Data is missing for change detection setsize 3 and 5 (letters) session 12.

Lost and excluded testing data

Noted below are instances where testing data was excluded prior to the 3 SD criteria. Data from participants 1007 and 1037 were included only in the first 3SD cleaning procedure.

- 1000: At pre-test, participant made too many errors on Trails B that the experimenter was unable to correct in time.
- 1000: At pre-test, Dodge was accidentally run for 9 minutes.
- 1001: Pre-test data for Visual Search/25 Boxes are missing.
- 1004: At pre-test, Dodge was accidentally run for 17 minutes.
- 1005: Post-test data for Form Boards are missing.
- 1008: Pre-test data for Form Boards are missing.
- 1009: At post-test, Dodge was accidentally run for 20 minutes.
- 1011: At post-test, the participant continued past level 1 of Dodge unsupervised and the timer was started at level 6.
- 1012: At pre-test, the data was not saved for the Dodge game.
- 1012: At post-test, the subject reported inaccurate feedback during the TSDT; no responses were being logged during part of the TSDT (likely keyboard/special key issue).
- 1016: At pre-test, data was not saved for the Dodge game.
- 1018: At pre-test, music from Dodge was left on while the participant completed Control Tower.
- 1030: The post-test data for Dodge is missing, most likely not saved by the experimenter.
- 1041: At pre-test, participant didn’t understand instructions during the first list of Paired Associates. Data not analyzed.
- 1043: At pre-test, the participant continued past level 1 of Dodge unsupervised and the timer was started at level 2.
- 1043: At post-test, there were no data recorded for this subject’s performance during Trail Making.
- 1046: At post-test, the participant did not reach some of the dots in the Trail Making test.
- 1048: At post-test, cognitive testing session 2 (instead of session 3) was accidentally administered after the MRI session. Sessions 3 and 1 were not completed until 19 days after post-testing session 2. Data from these last two testing sessions were discarded from analyses due to the time elapsed after training.
- 1100: At pre-test, music from Dodge was left on while the participants completed Control Tower.
- 1100: At post-test, the subject missed the last dot on Trails A.
- 1104: At pre-test, data from Dodge game were not saved.
- 1105: At pre-test, the experimenter switched the VSTM key presses when giving instructions to the participant.
- 1111: At pre-test, the participant had switched the VSTM key presses (switched meanings of the keys).
- 1115: At pre-test, music from Dodge was left on while participant completed the TSDT.
- 1119: At pre-test, the subject’s phone rang during the Trail Making test, during which he made 2 errors and was likely distracted.
- 1119: At post-test, participant misunderstood directions for the Paired Associates. Data not analyzed.
- 1120: Pre-test data from late level performance in Visual Search/25 Boxes (both runs) is missing, potentially restarted game at end of early levels.
- 1127: At post-test, the Matrix Reasoning task stopped responding and the screen went blank with approximately 3 minutes left into the task.
- 1129: At post-test, participant misunderstood how to do Paired Associates. Data not analyzed.
- 1132: At post-test, Operation Span crashed approximately halfway through due to a run time error.
- 1133: Pre-test i-Position data are missing.
- 1135: At post-test, only a .txt file from Operation Span was generated. The recovered e-dat file was not complete.
- 1149: At pre-test, the sound was muted for the Anti-Saccade test.
- 1149: At post-test, the fire alarm went off in the middle of Trails A and B.
- 1149: Post-test i-Position data files are missing.
- 1150: At pre-test, participant misunderstood directions while answering first list of Paired Associates. Data not analyzed.
- 1151: Pre-test data are missing (likely not saved) for Dodge.
- 1151: Post-test data are missing for Trails B.

Other Session Notes

Noted below are instances where data included in analysis was collected under less than ideal or atypical circumstances.

- 1001: At post-test, MATLAB crashed a few trials into VSTM. The task was restarted.
- 1002: At pre-test, participant was reminded not to use cell phone during VSTM.
- 1003: At pre-test, completed Pattern Comparison sheet two with 4 extra seconds. Experimenter used 13 out of the 15 of the correct and attempted items to estimate actual responses in 30 seconds.
- 1009: At pre-test, experimenter stopped the TSDT (subject didn’t understand the task), explained the instructions, and restarted the task.
- 1013: At pre-test, participant realized she was using the wrong keys for VSTM about 5 trials into the task, so it was restarted.
- 1013: At pre-test, participant realized that headphones were not connected during the tutorial for Control Tower. Headphones were on for the rest of the task.
- 1015: At post-test, the data was not saved for the Dodge game. However, the experimenter noted that the subject was at level 12 when the time ran out. For analysis, we used level 11 as the maximum level passed.
- 1015: At pre-test, was reminded not to check their phone during VSTM.
- 1015: At pre-test, restarted halfway through Number Search because participant thought Restart would take her to next level. Experimenter ran through Number search twice after this. Data from last two complete runs was analyzed.
- 1017: At pre-test, accidentally stopped after one minute during Digit Symbol, but started where she left off for the last minute.
- 1024: At pre-test, the single n-back task was restarted at the participant’s request because participant was confused about when to begin responding.
- 1024: At pre-test, TSDT crashed due to a pop-up window and the participant completed the task on a different computer.
- 1026: At pre-test, participant misunderstood instructions for the letter task in Control Tower, but this was corrected before the task began.
- 1027: At pre-test, Dodge and Number Search were restarted to review instructions.
- 1029: At pre-test, the participant mentioned that she had experience with the Running Span, Symmetry Span, and Operation Span, and that she had a encountered a task similar to Control Tower (though noted that the version in the current study was more complicated).
- 1029: At post-test, restarted Anti-Saccade task after the first practice because the volume was too loud.
- 1031: At pre-test, started skipping items during Digit-Symbol, but was corrected immediately.
- 1031: At post-test, accidentally exited the single n-back task. Task was restarted.
- 1031: At post-test, subject repeated Number Search game because participant clicked to play again before experimenter could save the data.
- 1035: At pre-test, Number Search was not saved properly (only round 1) so the subject completed Number Search again at the end of the session (two rounds). The output from all three rounds was analyzed.
- 1038: At post-test, i-Position crashed a few minutes after the task began, so the task was restarted.
- 1040: At pre-test, started Digit Symbol before experimenter was finished with directions. First two items were discarded, experimenter started timing after that.
- 1040: At pre-test asked a question ~25 seconds into the first sheet of Pattern Comparison. Experimenter paused timer to answer the question and timed the last 5 seconds.
- 1041: At pre-test, the experimenter said “morning” instead of “evening” during the first telling of the second story. Neither item was recalled.
- 1042: At post-test, subject completed TSDT and notified experimenter that music from Dodge was still playing. TSDT was restarted (including practice).
- 1048: At pre-test, repeated the single n-back task, since it crashed about halfway through the first run.
- 1101: The version of Control Tower used at pre-test was accidentally administered at post-test.
- 1102: At pre-test, output from first run of Number Search data was not saved, so data is only output from second run.
- 1103: At pre-test, Letter Sets ended early due to a pop-up window. Task was repeated and data from this repeated instance was analyzed.
- 1106: At pre-test, timer was accidentally stopped during Trails B (34.1 seconds). Experimenter estimates that completion time was 39-40 seconds—data entered as 39.5 seconds.
- 1107: At pre-test, i-Position crashed twice after the first experimental trial, so participant completed task on a different computer.
- 1108: At post-test, participant was reminded not to use cell phone during VSTM.
- 1109: At post-test, participant misunderstood instructions for Anti-Saccade and task was restarted three times. Data from the last instance was analyzed.
- 1111: At pre-test, participant completed second sheet of Letter Comparison for 45 seconds. Experimenter used ~2/3 of the total correct and attempted to estimate the score at 30 seconds.
- 1111: At pre-test, participant was asked to put phone away during the breaks in TSDT.
- 1112: At pre-test, participant completed the first sheet of Pattern Comparison upside down.
- 1115: At post-test, only one run of Number Search was saved.
- 1117: At pre-test, Paper Folding crashed after 5 trials. Participant restarted and completed the task on a different computer.
- 1119: At pre-test, VSTM and TSDT were restarted after few trials to review instructions.
- 1120: At pre-test, TSDT was restarted in the middle of run1 to review instructions.
- 1120: At pre-test, the participant was reminded not to use phone during Control Tower.
- 1121: At pre-test, passed bold line marking end of practice in Digit Symbol. Experimenter erased answers to non-practice items and started timing with participant starting after the bold line.
- 1121: At pre-test, mouse started malfunctioning near the end of the i-Position test. There was no time to repeat the test, so participant redid i-Position task at the end of the second testing session.
- 1124: At post-test, participant completed 3 rounds of Number Search due to clicking Restart, but only two rounds were saved.
- 1125: At pre-test, TSDT was restarted because experimenter forgot headphones and reviewed instructions.
- 1126: At post-test, a phone rang while subject was performing Logical Memory.
- 1127: At post-test, i-Position crashed during the first practice problem and experimenter restarted the program. The program crashed again at the beginning of the real task, so program was rerun (including practice).
- 1128: At pre-test, participant started skipping around during Digit Symbol, but was corrected.
- 1129: At post-test, participant inadvertently hit a key that ended the Psychomotor Vigilance Task (Vigilance Counter). The experimenter administered the Symmetry Span and then repeated the PVT.
- 1132: At pre-test, Dodge music was on during start of TSDT. TSDT crashed and reran.
- 1132: At post-test, subject clicked straight through directions for Paper Folding without stopping to read them.
- 1134: At post-test, the Anti-saccade test was accidentally administered twice, with the first set of data overwritten.
- 1135: At pre-test, participant reported being confused about the single n-back task after it was run (fully). The whole task was repeated.
- 1135: At post-test, participant accidentally closed Running Span and re-did the task.
- 1136: At post-test, participant needed to be reminded during i-Position that he is to click each symbol and count aloud.
- 1137: At post-test, Attentional Blink was restarted after the first trial due to confusion about the instructions.
- 1141: At pre-test, experimenter forgot to tell participant that they could use paper and pencil for Paper Folding.
- 1142: At pre-test, another experimenter walked into the room during Letter Comparison and may have distracted the participant.
- 1142: At pre-test, i-Position crashed after the first trial experimental trial and was restarted.
- 1144: At pre-test, i-Position was run as part of session 2 since time ran out during session 1.
- 1145: At post-test, participant did not complete i-Position practice.
- 1150: At post-test, i-Position crashed soon after participant started experimental trials. Task was restarted, but practice was not run for this second instance.
